# A Nutrient-Driven tRNA Modification Alters Translational Fidelity and Genome-wide Protein Coding across an Animal Genus

**DOI:** 10.1371/journal.pbio.1002015

**Published:** 2014-12-09

**Authors:** John M. Zaborske, Vanessa L. Bauer DuMont, Edward W. J. Wallace, Tao Pan, Charles F. Aquadro, D. Allan Drummond

**Affiliations:** 1 Department of Biochemistry and Molecular Biology, University of Chicago, Chicago, Illinois, United States of America; 2 Department of Molecular Biology and Genetics, Cornell University, Ithaca, New York, United States of America; 3 Department of Human Genetics, University of Chicago, Chicago, Illinois, United States of America; Fred Hutchinson Cancer Research Center, United States of America

## Abstract

Natural selection favors efficient expression of encoded proteins, but the causes, mechanisms, and fitness consequences of evolved coding changes remain an area of aggressive inquiry. We report a large-scale reversal in the relative translational accuracy of codons across 12 fly species in the *Drosophila*/*Sophophora* genus. Because the reversal involves pairs of codons that are read by the same genomically encoded tRNAs, we hypothesize, and show by direct measurement, that a tRNA anticodon modification from guanosine to queuosine has coevolved with these genomic changes. Queuosine modification is present in most organisms but its function remains unclear. Modification levels vary across developmental stages in *D. melanogaster*, and, consistent with a causal effect, genes maximally expressed at each stage display selection for codons that are most accurate given stage-specific queuosine modification levels. In a kinetic model, the known increased affinity of queuosine-modified tRNA for ribosomes increases the accuracy of cognate codons while reducing the accuracy of near-cognate codons. Levels of queuosine modification in *D. melanogaster* reflect bioavailability of the precursor queuine, which eukaryotes scavenge from the tRNAs of bacteria and absorb in the gut. These results reveal a strikingly direct mechanism by which recoding of entire genomes results from changes in utilization of a nutrient.

## Abstract 2

Use of the nutrient queuine to modify tRNA anticodons can change the accuracy of certain codons during protein synthesis, resulting in evolutionary recoding of fruit fly genomes.

## Author Summary

Ribosomes translate mRNA into protein using tRNAs, and these tRNAs often translate multiple synonymous codons. Although synonymous codons specify the same amino acid, tRNAs read codons with differing speed and accuracy, and so some codons may be more accurately translated than their synonyms. Such variation in the efficiency of translation between synonymous codons can result in costs to cellular fitness. By favoring certain coding choices over evolutionary timescales, natural selection leaves signs of pressure for translational fidelity on evolved genomes. We have found that the way in which proteins are encoded has changed systematically across several closely related fruit fly species. Surprisingly, several of these changes involve two codons both read by the same tRNA. Here we confirm experimentally that the anticodons of these tRNAs are chemically modified—from guanine to queuosine—*in vivo*, and that the levels of this modification in different species track the differences in protein coding. Furthermore, queuosine modification levels are known to change during fruit fly development, and we find that genes expressed maximally during a given developmental stage have codings reflecting levels of modification at that stage. Remarkably, queuosine modification depends upon acquisition of its precursor, queuine, as a nutrient that eukaryotes must obtain from bacteria through the gut. We have thus elucidated a mechanism by which availability of a nutrient can shape the coding patterns of whole genomes.

## Introduction

Because the genetic code maps the 64 possible nucleotide-triplet codons to only 20 amino acids and three stop signals, proteins can be coded in multiple ways in the genome using different sets of synonymous codons. Despite specifying the same amino-acid sequence, the particular coding employed can alter fitness, sometimes dramatically [1], resulting in the highly nonrandom codings found in extant genomes [2],[3]. Although selection has long been thought to only weakly shape variation in synonymous codings, very recent evidence from *Drosophila* indicates a much stronger potential role for selection [4].

Selection acts in a host of different ways to constrain the evolutionarily viable set of protein codings, with most constraints imposed on aspects of gene expression. The charged tRNA molecules that physically embody the genetic code, bearing a triplet anticodon on one end and an amino acid at the other, read codons with differing speed and accuracy [5],[6] arising from their cellular abundances and kinetic properties.

Recent work has uncovered an ever-multiplying panoply of potential mechanisms by which codon choice alters fitness. Codon choice influences the stability of mRNA secondary structures [7],[8] and reduced stability associates with higher protein production, consistent with higher rates of translational initiation [9],[10]. Slowly translated codons may induce ribosomal pauses necessary for proper protein folding and targeting [11], or regulate the entry of ribosomes into coding sequences in ways which limit jamming [12]. Adding to the complexity are mechanisms which constrain synonymous codon choice due to pressures on other processes, such as mutational biases and selection for efficient splicing [13].

All of these effects remain limited in their ability to explain the biased use of certain codons over their synonyms at the genome scale [14]. Two mechanisms remain dominant: selection on translational speed, and selection on translational accuracy.

Across widely diverged bacterial species, shorter generation times correlate with increases in total tRNA and ribosomal RNA (rRNA) copy number and elevated preferential usage of particular codons in high-expression genes [15]–[17]. These trends constitute evidence for selection acting to speed ribosomal transit across transcripts. Increased speed reduces the density of ribosomes on transcripts, thus raising the proportion of unbound ribosomes, which accelerates the translation initiation rate and, finally, overall protein production rate [18]. Consequently, selection for increased growth rate favors coding sequences that cause rapid elongation rates.

Evidence for selection on speed remains sparse in multicellular organisms [19], and recent work has failed to find systematic codon-dependent ribosome velocity differences correlated to codon usage [19]–[21]. Selection on speed may be of reduced importance for animals [17], whose developmental processes sharply reduce the coupling between fitness and the cell doubling rate.

By contrast, natural selection to improve translational accuracy has been demonstrated in organisms ranging from bacteria to humans [22]–[24]. Amino acid errors at the ribosome, estimated to occur in roughly one out of every five average-length proteins [25], may cause loss of function [22] or cytotoxic misfolding [24],[26]. Consequently, coding sequences which reduce such errors, and reduce their impact on folding and function, will be favored by selection. Selection against mistranslation-induced misfolding suffices to generate major patterns of accuracy-driven codon usage observed from bacteria to humans [24].

Akashi introduced a clever method to isolate selection on translational accuracy [22], which has since been widely applied [23],[24],[27]. Akashi's test quantifies the tendency of particular codons, such as those corresponding to abundant tRNAs, to be found encoding amino acid sites that are sensitive to substitution, such as those conserved over evolutionary time, where errors in translation are likely to be most costly [22].

The use of tRNA abundance estimates to predict which codons will be most efficiently translated has become commonplace. A standard approach predicts tRNA abundances from modestly correlated but more readily measurable genomic tRNA gene copy numbers [12],[28],[29], and designates codons “optimal” or “preferred” if they are predicted to be read by the most-abundant tRNAs. However, tRNAs are heavily chemically modified, often in the anticodon [30], making assignments of which tRNA reads which codon nontrivial. As a relatively well-known example, a eukaryotic tRNA with a genomically encoded anticodon 5′-AGC-3′ might be naively predicted to bind and read the alanine codon GCU more readily than the synonym GCC. Instead, such tRNAs generally have their 3′-adenine modified post-transcriptionally to inosine (I) by tRNA-adenosine deaminases, yielding 5′-IGC-3′, which binds GCC more strongly than GCU [31]. Accounting for these modifications substantially improves the correlation between genomic codon usage and levels of corresponding tRNA [17],[32].

Codons corresponding to the most-abundant tRNAs are often assumed to be read more rapidly and more accurately. Consideration of the kinetics of translation, however, indicates that this need not be true [17]: codons read by high-abundance tRNAs may also be misread by high-abundance near-cognate tRNAs, reducing their accuracy [33].

These studies reveal the surprising richness of selection on protein coding across the tree of life. Both speed and accuracy selection play substantial roles, although major questions remain about the relative strength of selection on these traits [20]. Developing a mechanistic answer to the question of what determines genome-wide protein coding requires synthesizing translation kinetics, tRNA biochemistry, mutational processes, gene expression, population genetics, organism life-history traits, and systems-level pressures on organism fitness. Changes in coding between orthologous proteins are easy to find, but only between organisms that have diverged on many if not all of these contributing factors.

Here we report the discovery and mechanistic illumination of whole-genome recoding restricted to the well-studied drosophilids (*Drosophila melanogaster* and its relatives), in which codon choice has been thought to be highly conserved [34]. We develop a novel measure for selection on translational accuracy, based on Akashi's insight, which reveals a large-scale, phylogenetically coherent reversal in the relative accuracy-driven fitness benefit of multiple codons over their synonyms. To explain this reversal, we hypothesize that levels of a known tRNA modification in the anticodon, guanine to queuosine, change across species. We detect this quantitative change in queuosine modification directly in tRNAs of four species by electrophoretic separation, finding that modification levels vary exactly opposite published predictions. We then predict, and verify, that because queuosine modification levels change throughout *D. melanogaster* development, the accuracy-driven codon usage of genes expressed at different developmental stages should covary with the modification level much as they do across the phylogeny. We propose a kinetic model to explain how changes in queuosine modification suffice to reverse the relative accuracy of synonymous codons, while preserving their relative speed. Surprisingly, queuosine modification is known to be largely determined by intake of the precursor nutrient queuine, which animals solely acquire from bacteria, providing a remarkably simple pathway for nutrient availability to alter genome-scale protein coding.

## Results

Akashi's insight allows use of equilibrium frequencies of codons, and their conservation across species, to estimate the average population-scaled selective advantage due to a codon change that is attributable to translational accuracy selection (see Methods). The essential procedure, closely following Akashi's, is to estimate the population-scaled difference in fitness between a synonymous change at a conserved site (where the same amino acid is preserved in all 12 drosophilid species) compared to the same change at a variable site (all other sites) within the same coding sequence. We term this scaled selective advantage the *Akashi selection score*. Because it derives entirely from within-gene information, this score is unaffected by between-gene differences in expression level, function, and so on; to the extent that variation in these features affects selection for accuracy, the Akashi selection score will tend to underestimate overall selection on accuracy. However, it gains specificity for the mechanism of selection, a unique and powerful feature.

**Figure 1:**
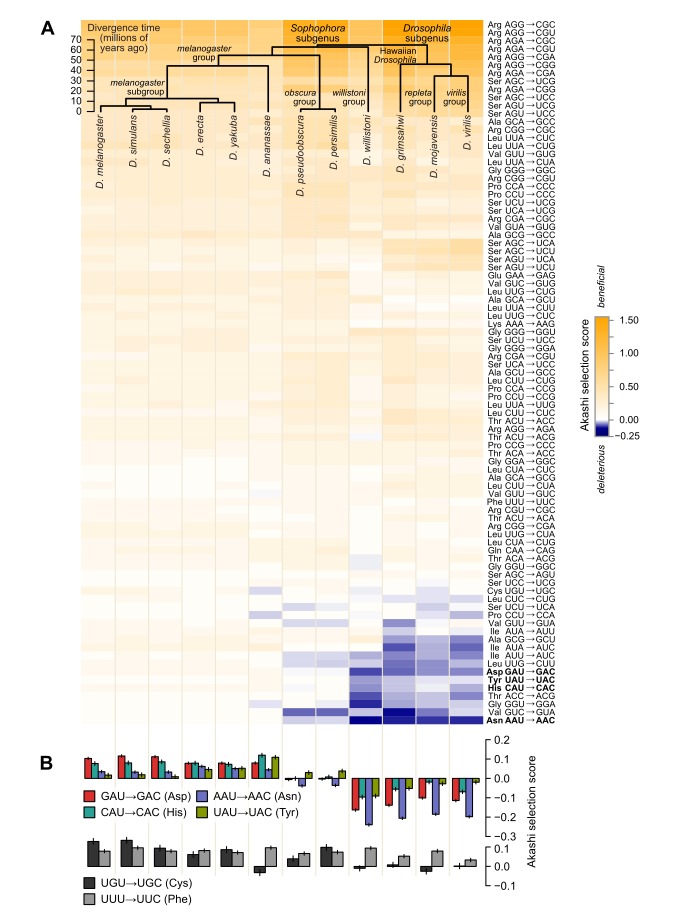
Relative codon translational accuracy shifts coherently across the drosophilid phylogeny. (A) Overview of Akashi selection scores, the average population-scaled fitness difference between a synonymous codon change at a conserved amino acid site compared to the same change at variable site within the same gene. Selection scores are symmetric, such that score(codon X to codon Y) = −score(Y to X). All possible synonymous codon-to-codon pairs are shown, with the order of X and Y chosen such that the *D. melanogaster* values are positive. (B) Akashi selection scores for all 2-fold-degenerate C/U-ending codons. Error bars indicate 95% confidence intervals.

We determined Akashi selection scores for 5,182 genes present in 1∶1∶‥∶1 orthologs across 12 sequenced drosophilid species (Figure 1A), an analysis covering 13.7 million conserved and 8.2 million variable sites in *D. melanogaster* with similar numbers in the other species. The Akashi selection scores are symmetric, in that the benefit gained from changing one codon to another is equal to the cost of reversing this change. To facilitate comparisons between species, we orient the codon changes such that the *D. melanogaster* scores are all positive, indicating an accuracy benefit in changing one codon into another, and sort the rows from most-beneficial to most-deleterious (Figure 1A) or by amino acid (Figure S1). For *D. melanogaster*, codons conferring the greatest accuracy benefit in our mechanistically specific analysis closely match those reported to be “favored” or “optimal” in previous analyses (Table S1), confirming previous results using a structural approach to detect selection on accuracy [23].

Codon usage across 12 sequenced species of drosophilids has been examined in multiple studies, always with the conclusion that codon usage is largely stable, with *D. willistoni* the notable exception [34]–[36]. If codon usage were largely stable, our data would show nearly uniform benefit preserved from *melanogaster* across the phylogeny to *virilis*. But instead, a strikingly different cross-species portrait of codon usage emerges compared previous studies. The Akashi selection scores of many codon changes shift from beneficial to deleterious, and *D. willistoni* forms part of a coherent trend which spans the *Drosophila* subgenus (Figure 1A; Table S2). Among the amino acids showing the strongest and most consistent shifts, evolutionarily conserved aspartates, histidines, asparagines, and tyrosines are more often encoded by the codon NAC (where N = G, C, A, or U, respectively) in the *melanogaster* subgroup, exemplified by *D. melanogaster* itself, and by NAU in the *Drosophila* subgenus, exemplified by *D. virilis* (Figure 1B). The remaining two-codon C/U-ending families, encoding cysteine and phenylalanine, shift modestly or not at all (Figure 1B). These results therefore expose a previously unreported cross-species shift in codon usage linked specifically to selection on translational accuracy.

**Figure 2:**
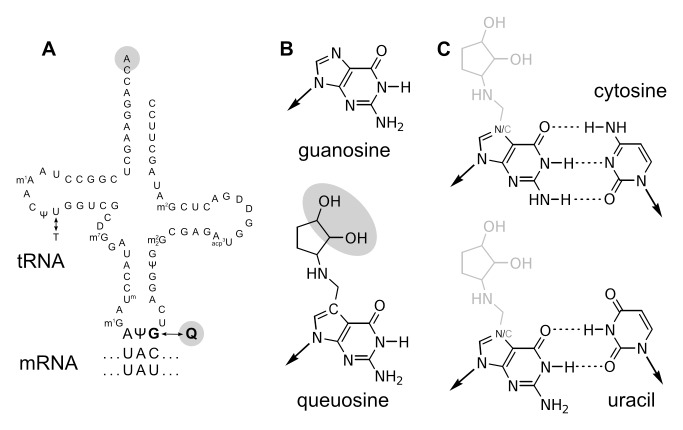
Queuosine modification alters features of tRNA anticodons. (A) The sequence of *D. melanogaster* tRNA^Tyr^ (after Suter and colleagues [62]) shows modification of guanosine (G) to queuosine (Q) in the anticodon position corresponding to the third-position (wobble) base of the codon. Positions of *cis*-diol moieties are highlighted in gray. (B) Guanosine (top) and queuosine (bottom); *cis*-diol highlighted in gray. Arrows point toward the primary ribose moiety which is not shown. (C) Guanosine and queuosine binding cytosine (C, top) and uracil (U, bottom).

Because tRNAs bearing a single genomically encoded anticodon read both codon synonyms in all 12 species (Table S3) [34],[37], changes in tRNA gene copy number or tRNA gene expression cannot explain the reversals. However, in *D. melanogaster*, as in most other organisms, the anticodons of the tRNAs that read these four amino acids are partially modified *in vivo* by tRNA-guanine transglycosylase (TGT) from guanosine (G) to queuosine (Q) in the 5′ (wobble-binding) position (Figure 2A) [38].

In eukaryotes, Q modification of tRNA depends on scavenging the precursor nutrient queuine from the anticodons of bacterial tRNAs [39] obtained either by feeding or from gut microbiota [40]. Despite decades of study, the function of the queuosine modification remains unclear, as do its biochemical effects. In normally fed *D. melanogaster*, levels of Q modification vary over the course of development [38],[41], dropping to their lowest point in third-instar larvae and peaking in adults [38]. The restriction of Q modification to the anticodon strongly suggests a role in translation. *In vitro*, Q-modified tRNA bound C-ending triplets more stably than U-ending triplets [31]. Structural studies report that Q-tRNA and G-tRNA have similar codon-recognition properties [42], as might be naively expected because the ribose moiety differentiating Q from G does not involve the codon-recognizing portion of the nucleoside [43] (Figure 2B). *Drosophila* tRNA^His^ injected into *Xenopus* oocytes translates NAU more than NAC when Q-modified and NAC when unmodified [44]. Q-modified tRNA has a higher apparent affinity for ribosomes in bacterial and eukaryotic systems [44],[45].

### Direct Measurement of a Coherent Shift in tRNA Modification across the Drosophilids

In pioneering work, Powell and colleagues hypothesized that elevated Q modification might explain the unusual codon usage they observed in *D. willistoni* under the assumption that Q-modified tRNA preferentially reads U-ending codons [46]. Measurements of TGT gene expression levels as proxies for Q modification levels produced ambiguous results [47].

**Figure 3:**
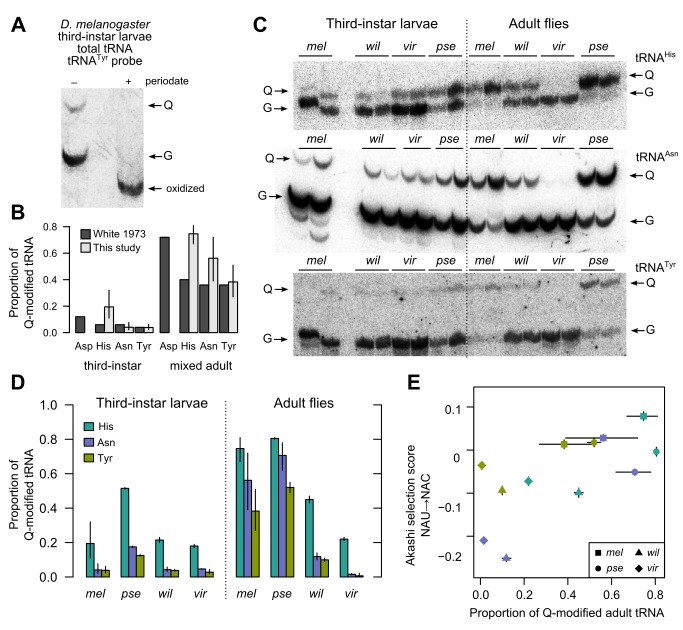
Queuosine tRNA modification covaries with relative codon accuracy across the drosophilid phylogeny. (A) Northern blot of total *D. melanogaster* third-instar larval tRNA using tRNA^Tyr^-specific probe resolves two major bands (left) after electrophoretic separation on an acryloyl aminophenylboronic acid gel (APB gel); when *cis*-diols are oxidized with periodate, tRNA runs as a single band (right). (B) APB gel measurements of Q modification produce similar stage-specific results to an independent method in *D. melanogaster*. (C) Separation of total tRNA from four species at two developmental stages by APB gel followed by Northern blotting using probes specific for each species' tRNA^His^, tRNA^Asn^, and tRNA^Tyr^ reveals shifts in Q modification (*mel*, *D. melanogaster*; *pse*, *D. pseudoobscura*; *wil*, *D. willistoni*; *vir*, *D. virilis*). (D) Quantification of the data in (C) (cf. Data S1); error bars show standard error in measurement. (E) The Akashi selection scores for NAU to NAC, over all genes, track the proportion of Q-modified tRNA in adult flies across species. Colors as in (D). Bars show standard error of the mean (SEM) for modification levels and 95% confidence interval for selection scores. Spearman rank correlation *r* = 0.61, *p*<0.05 for raw values, and *r* = 0.73, *p*<0.01 after subtracting means from each synonymous family.

We therefore employed a method to quantify Q modification levels directly starting from total RNA. *Cis*-diol moieties, such as the 3′ ribose of every tRNA, slow migration through gels composed of polyacrylamide covalently linked with *N*-acryloyl-3-aminophenylboronic acid (APB) [48]. Consequently, queuosine's additional ribose moiety (Figure 2B) slows Q-tRNA migration relative to G-tRNA, producing two bands on an APB gel [48]. This differential migration can be eliminated by oxidizing the ribose *cis*-diols with periodate, producing a single faster-running band [48]. We confirmed these expected effects by Northern blotting of total RNA from *D. melanogaster* with a probe specific to tRNA^Tyr^ (Figure 3A). Subsequent quantification of Q modification in *D. melanogaster* tRNA^His^, tRNA^Tyr^, and tRNA^Asn^ confirmed that Q-tRNA abundance is low in third-instar larvae and rises until roughly half of these tRNAs are modified in adult flies, consistent with results of a previous study using an independent, chromatography-based method to quantify Q modification (Figure 3B) [38]. We were not able to isolate separate Q- and G-tRNA^Asp^ bands on APB gels, likely due to a secondary mannosyl-queuosine modification [49].

We then quantified Q- and G-tRNA^Tyr^, -tRNA^Asn^, and -tRNA^His^ in third-instar larvae and adult flies of *D. pseudoobscura*, *D. willistoni*, and *D. virilis*, species which span the drosophilid phylogeny. Substantial differences were apparent, with *D. willistoni* and *D. virilis* showing lower levels of Q modification than *D. pseudoobscura* and *D. melanogaster* for each tRNA species. Modification levels and between-species differences were greater in adults than in larvae (Figure 3D). TGT gene expression poorly predicted modification levels (Figure S2).

Queuosine tRNA modification in adult flies, but not larvae, shows a significant and positive correlation with an accuracy-driven selective advantage favoring C- over U-ending codons, quantified by the Akashi selection score, across all codons and species (adults, Spearman *r* = 0.61, *p*<0.05, Figure 3D; larvae, *r* = 0.05, *p* = 0.86, not shown). Importantly, the relationship between Q modification and Akashi selection score in adults is positive not just in aggregate, but also within each codon family (minimum Spearman *r* = 0.6 for His, Asn, and Tyr, with *p*>0.05 due to small sample size). Indeed, variation in the characteristic level of modification in, and selection upon, each synonymous family will tend to spuriously reduce the observed relationship between Q modification and Akashi selection score. After subtracting means from each family, the overall Spearman correlation across all families is *r* = 0.73, *p*<0.01, indicating that Q modification suffices to explain more than half the variation in selection scores across species.

### Accuracy-Driven Selection Shifts with Q Modification across Development

**Figure 4:**
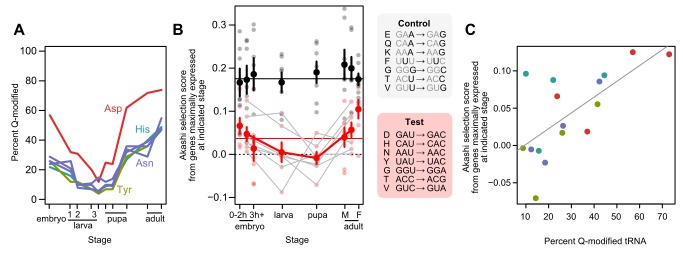
Queuosine tRNA modification covaries with relative codon accuracy across developmental stages in *D. melanogaster*. (A) Data from White and colleagues shows tRNA modification over the course of development, including three instar larval stages. (B) Akashi selection scores derived from genes maximally expressed at each of six developmental stages (0- and 3-hour embryos, larva, pupa, and adult males [M] and females [F]). Values from pooling both embryo stages and both adult stages are also shown between the two pooled groups. Test codons (red points) are those showing the strongest accuracy-selection shifts across the drosophilid phylogeny, hypothesized to be driven by queuosine tRNA modification (cf. Figure 1). Control codons differ from test codons by a single nucleotide, highlighted. For test codons, the pooled embryo versus larva and versus pupa stage, and the larva and pupa stage versus pooled adult stages, are significant at *p*<0.05 (one-tailed Wilcox signed-rank test). For control codons, all contrasts are insignificant using identical tests (*p*>0.2). Median values for test and control codons across all stages are shown as solid horizontal lines. Error bars show standard error on the mean. Gray lines show changes across each synonymous test-codon pair, and a solid red line tracks the mean value across pooled-embryo, larva, pupa, and pooled-adult stages. (C) Akashi selection scores for codons cognate to Q-modified tRNAs correlate with the Q-modification levels of these tRNAs. Modification levels for the three Asn isoaccepting tRNAs were averaged, and mean values for embryo, larva, pupa, and adult were used. Colors as in (A). Gray line shows linear best fit. Spearman rank correlation *r* = 0.61, *p*<0.02 for raw values, and *r* = 0.63, *p*<0.01 after subtracting means from each synonymous family.

Based on these cross-species correlational results, we hypothesized that Q modification alters relative codon accuracies, creating a signature of selection that we can observe using Akashi selection scores. A serendipitous opportunity to test this causal hypothesis arises from the observation that levels of Q modification vary across development within a species (Figure 4A, results from White and colleagues [38]). If the level of Q modification alters relative codon accuracy, then genes expressed at their highest levels in a particular developmental stage should experience accuracy selection modulated primarily by the level of Q modification at that stage. That is, we predict that specific codon substitutions—the ones which reverse their Akashi selection scores across species as adult-stage Q modification drops—will change their selection coefficients in the same direction during *D. melanogaster* developmental stages where Q modification drops. To test this prediction, we determined the Akashi selection scores using non-overlapping sets of genes maximally expressed at several *D. melanogaster* developmental stages similar to those in the White and colleagues' study (early/late embryo, larva, pupa, adult male/female) [50],[51]. We performed statistical tests on gene sets pooled into four categories: maximal expression in embryo, larva, pupa, or adult flies.

We focused on the seven synonymous codon pairs showing the strongest changes in Figure 1: four pairs encoding amino acids read by Q-modified tRNAs, and three pairs showing shifts at least as strong (Figures 1A and 4B). As predicted, these seven pairs showed a systematic shift in Akashi selection scores from moderately positive in favor of the C-ending codon during the embryonic stage, where Q modification is elevated, to near zero or negative (favoring the U-ending codon) during the larval and pupal stage, where Q modification is lowest, rising again to strongly positive in adults, where Q modification is highest (Figure 4; Table 1). As with variation across species (Figure 3), variation in Akashi selection scores for Asp, His, Asn, and Tyr codons over development correlates with the modification levels of the corresponding tRNA species (Figure 4C, three tRNA^Asn^ isoacceptor levels averaged) with *r* = 0.61, *p*<0.02. As noted before, differing mean levels of selection and modification in each family add unwanted noise, and also as before, subtracting means from each family yields a stronger correlation, *r* = 0.63, *p*<0.01.

**Table 1:** Akashi selection scores for test and control codon pairs.

Amino Acid	Change	Developmental Stage							
		0–2 h Embryo	3–16 h Embryo	Embryo	Larva	Pupa	Adult	Adult Male	Adult Female
Pairs shifting strongly across phylogeny									
D	GAU→GAC	0.1108	0.1500	0.1250	0.0659	0.0186	0.1222	0.1238	0.1181
H	CAU→CAC	0.1516	−0.0226	0.0877	0.0961	−0.0072	0.0941	0.0713	0.1644
N	AAU→AAC	0.0162	0.0444	0.0263	−0.0053	−0.023	0.0857	0.0731	0.1233
Y	UAU→UAC	0.0628	−0.0697	0.0169	−0.0037	−0.0707	0.0553	0.0516	0.0676
G	GGU→GGA	0.0508	−0.0674	0.0066	−0.0877	0.0499	0.0275	0.0382	−0.0036
T	ACC→ACG	0.0228	0.0354	0.0274	−0.0294	0.0003	0.0008	−0.0351	0.1079
V	GUC→GUA	0.0480	0.0251	0.0402	−0.0015	−0.0269	0.0125	−0.0435	0.1546
Control codon pairs									
E	GAA→GAG	0.2448	0.3013	0.2651	0.2325	0.2343	0.1716	0.1758	0.1590
Q	CAA→CAG	0.1143	0.0924	0.1067	0.1447	0.0976	0.1716	0.1496	0.2302
K	AAA→AAG	0.1265	0.1522	0.1352	0.1935	0.2035	0.1750	0.1731	0.1803
F	UUU→UUC	0.0449	0.0329	0.0410	0.0801	0.1178	0.0974	0.0829	0.1436
G	GGG→GGC	0.2854	0.2261	0.2626	0.1980	0.2573	0.2943	0.3378	0.1627
T	ACU→ACC	0.1283	0.2072	0.1559	0.0928	0.1595	0.1983	0.2212	0.1311
V	GUU→GUG	0.2223	0.2889	0.2451	0.2268	0.2622	0.2888	0.3171	0.2118

Wilcoxon signed-rank tests indicated significant reductions in Akashi selection scores in genes expressed when Q is lowest (larva, pupa) compared to genes expressed when Q is highest (embryo, adult) (all four comparisons *p*<0.05). Comparisons between larva and pupa, and between embryo and adult, were not significant.

Most pairs individually follow the predicted pattern, though there are some exceptions. The benefit of CAC over CAU (His), for example, rises slightly from embryonic to larval stages before dropping markedly in pupa and rising again in the adult stages. Remarkably, though, we even observe reversals of relative codon accuracy selection during development: all Akashi selection scores are positive during the embryonic stage (mirroring the genome-wide average), yet more than half turn negative in the larval stage, and all but one (for Asp codons) switches sign twice between the embryo and adult stages. Asp, while always showing a benefit in favor of GAC over GAU, shows the predicted U-shaped change in selection scores mirroring the change in Q modification.

As a control, we examined the selection scores for seven codon pairs chosen to be as similar as possible to the seven shifting pairs examined above. Each of these pairs differed from one of the shifting pairs by a single nucleotide substitution, in the wobble position wherever possible (Figure 4B). These control pairs showed no significant changes across any of the developmental stages (Wilcoxon signed-rank test *p*>0.2 for all comparisons), demonstrating the specificity of the observed shifts in selection scores linked to Q modification.

Together, these results show the predicted shift in accuracy-driven codon usage during *D. melanogaster* development corresponding to changes in Q modification of tRNA. Higher levels of Q modification correspond to increasing use of C-ending over U-ending codons at sites encoding conserved amino acids. Because these predictions were made on the basis of cross-species changes in tRNA modification, and there is no other known connection between the developmental progression of *D. melanogaster* and the divergence of species across the phylogeny, we conclude that Q modification is likely to be the major cause of the changes in codon usage observed in both situations.

### A Kinetic Model Accounts for Modification-Driven Differences in Codon Accuracy

That Q modification correlates with usage of C-ending codons is perplexing because previous work in *D. willistoni* made the opposite prediction [47]. The idea behind that prediction was simple: assuming that G-tRNA translates C-ending codons more rapidly than U-ending codons, and observing that U-ending codons are used more frequently in *D. willistoni*, it makes sense to guess that Q-tRNA preferentially translates U-ending codons, and therefore that Q modification should rise in *D. willistoni*. So how is it possible for both G-tRNA and Q-tRNA to preferentially translate C-ending codons, but for selection to switch to favoring U-ending codons? Moreover, why do some codons that are not read by Q-modified tRNAs shift in their relative accuracy when Q modification changes?

We argue that substantial insight into these questions can be gained by examining the kinetic effects that modification may have on translational fidelity. In what follows, we present a model in which changes in Q modification alone suffice to cause the observed changes in relative accuracy, which selection would then act upon by recoding genes.

The selective changes observed in Figures 1 and 4 arise because of translational accuracy. Experimental work has established that accuracy is determined by tRNA competition [5], which can be quantified by the fraction of time a codon is translated by a cognate tRNA bearing the proper amino acid (the “right” tRNA) rather than a near- or non-cognate competitor tRNA bearing another amino acid (the “wrong” tRNA). Translation has many identifiably distinct kinetic steps, from initial binding to accommodation to proofreading to translocation, offering several places in which right and wrong tRNAs might differ and so alter their competition. Because it is not yet known in detail how queuosine tRNA modification alters any one of these steps, we concentrate on the overall rate of translation of a codon by a tRNA, which is a complex function of all kinetic steps.

The essential idea in the model detailed below is that the focal C-ending codons are translated rapidly, and mistranslated rapidly, by both right and wrong tRNAs, respectively, making their translation inaccurate unless the right tRNA is altered such that it overpowers the competitor. Q modification confers such strength. In the absence of Q modification, the U-ending codon, while read more poorly by the right tRNA, is more accurate because the competition from the wrong tRNAs is yet weaker. Thus, the presence of the modification alone is sufficient to determine which codon will be more accurately translated, and therefore favored by selection on accuracy.

**Figure 5:**
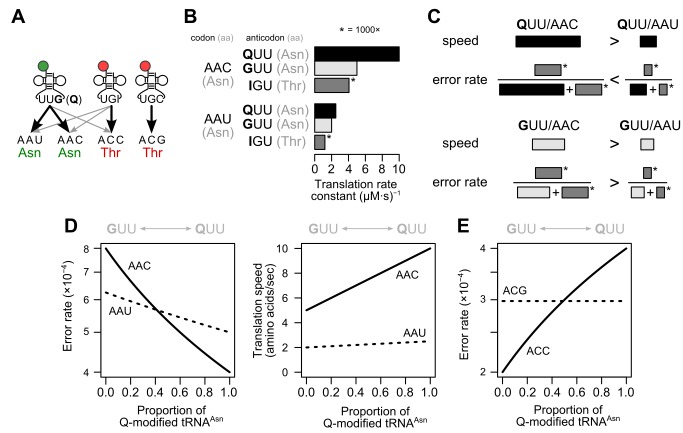
A kinetic competition model illustrates how Q modification alone can reverse relative codon accuracy. (A) Schematic representation of tRNA and codon relationships. Black lines represent cognate tRNA/codon relationships, and gray lines represent non-cognate (misreading) relationships. (B) A kinetic model produces rates for each tRNA reading the two asparagine codons AAC and AAU (cf. Listing S1). Misreading rates by tRNA^Thr^(IGU) are multiplied by 1,000 for visibility. The translation rate constant is proportional to the translation rate assuming equal tRNA concentrations, which we do for simplicity. (C) Graphical view of how rates given in (B) combine to produce speeds and error rates for each codon/tRNA pair. In the example, tRNA^Asn^(QUU) reads AAC faster and more accurately. In contrast, tRNA^Asn^(GUU) reads AAC faster, but AAU more accurately. (D) Quantitative error rates and translation speeds as a function of Q-modification in the model. (E) Modeled accuracy of the threonine codon ACC, which is assumed to be misread by tRNA^Asn^, changes with Q-modification, whereas ACG, which is not misread by tRNA^Asn^, does not, again resulting in a shift in relative accuracy.

To determine whether Q modification is sufficient to explain the reversal in relative codon accuracy within synonymous families, we constructed a simplified kinetic model of translation of a focal codon in which all tRNAs have the same molecular abundance (Methods) (Figure 5A; Listing S1) [52]. We focus on the asparagine codons AAU and AAC and their cognate tRNA^Asn^ (anticodon 5′-GUU-3′, for G-tRNA, or QUU, for Q-tRNA). The competitor near-cognate tRNA is threonine tRNA^Thr^(IGU), where I denotes inosine (see Introduction); we refer to this species as I-tRNA.

The model assumes that G-tRNA, Q-tRNA, and the near-cognate I-tRNA all have higher first-order rate constants for reading C-ending codons than for U-ending codons (Figure 5A), consistent with *in vitro* binding studies [31]. Q-tRNA is assumed to bind more rapidly than G-tRNA to any given codon, consistent with a higher affinity of Q-modified tRNA for ribosomes [45]. Finally, the relative rate of Q-tRNA reading C-ending over U-ending codons is assumed higher than for G-tRNA. Under these assumptions, the identity of the most accurately translated (lowest error rate) synonymous codon in a family can switch from C-ending to U-ending solely as a function of changes in queuosine modification (Figure 5B and 5C). This model generates error rates (between 10^−4^ and 10^−3^) and translation speeds (1–10 amino acids per second) matching physiological estimates (Figure 5C) [53],[54]. While the model's precise parameters are surely inaccurate, its value lies in showing that tRNA modification alone is capable of inducing an accuracy reversal under biologically plausible conditions.

The kinetic competition model offers a unique and intuitive explanation for why codons that are not normally read by a Q-modified tRNA nonetheless shift in accuracy when Q modification levels change: these codons are misread by Q-tRNA. If Q modification primarily increases tRNA affinity for ribosomes, then increased Q modification will reduce the accuracy of near-cognate codons due to misreading by Q-tRNA (Figure 5D). This accuracy reduction is detectable as a reduced Akashi selection score. Kinetically, accuracy reduction arises when we consider the inverse of the above problem: misreading of threonine ACC codons by G/Q-tRNA^Asn^ (which would properly read AAC/AAU codons). Given the apparent codon preferences of Q-modified tRNA, we can predict that ACC and ACU will be misread more often by Q-tRNA^Asn^ than will ACG, which is read by a separate tRNA, tRNA^Thr^(CGU). Consistent with this prediction, ACC is deleterious relative to ACG in the *melanogaster* subgroup where Q modification is highest, and beneficial in *D. virilis* where Q modification is nearly absent (Figure 1A; Table S2). Indeed, the relative benefits of A- or G-ending codons compared to U- or C-ending synonym change similarly for six amino acids (Gly, Thr, Val, Pro, Ser, and Leu) (Figure 1A; Table S2).

Most but not all observed codon-usage shifts can be explained by this kinetic model. The major exception is isoleucine, for which the A-ending codon AUA has an accuracy benefit over AUC/AUU in *virilis* but a cost in *melanogaster*. The isoleucine codon AUA is costly relative to AUU in every developmental stage except for larva, the lowest-Q stage, where the fitness cost becomes insignificant. This change mirrors the changes in accuracy benefit of these two codons in *D. virilis*, the lowest-Q species in our measurements, suggesting a link to the modification which is not captured by our kinetic model.

The kinetic model predicts that, unlike accuracy, the relative speed of codons always favors C-ending codons regardless of the level of Q modification (Figure 5B and 5C). That is, speed and accuracy selection can come into conflict dependent on the modification level, where one codon is more accurately translated but less rapidly translated than its synonym. If selection for speed were strong enough for a set of genes, those genes would show little or no accuracy-driven shift. A previous analysis found that genes encoding ribosomal proteins show consistent use of C-ending codons for His/Asn/Tyr across the phylogeny, but Asp codons shift in usage from C-ending to U-ending [18]. Because selection on speed favors increased production of ribosomes, ribosomal proteins may be expected to bear strong signatures of speed selection in addition to accuracy selection, making them unusually subject to speed/accuracy conflicts. We hypothesize that in most cases the speed benefit overwhelms the accuracy cost of C-ending codons in low-Q conditions for His/Asn/Tyr, but that accuracy costs outweigh speed benefits for Asp—perhaps because Asp codon mistranslation yields products that are particularly disruptive to ribosomal assembly or function. Our hypothesis illustrates the larger principle that the outcome of speed/accuracy conflicts can be amino-acid-specific, depending upon the consequences of speed and accuracy differences for each synonymous codon.

## Discussion

We find that entire genomes, under pressure for both accurate and rapid translation, have been recoded to maintain translational accuracy dependent on a tRNA modification. This modification varies across development, and the coding in genes expressed at different stages depends on the stage-specific modification level. Contrary to the common assumption that certain codons are “optimal” for translational speed and accuracy, we show how particular pairs of codons can reverse their relative accuracies while preserving their relative speeds. Our results provide evidence for multiple such speed/accuracy conflicts, building on the kinetic distinction between the translational accuracy and speed of codons articulated in previous studies [17],[33]. Going further, we show that a similar modification-dependent shift occurs during the developmental process of a single species, a striking example of the plasticity of translational fidelity. These results indicate that, if a codon is to be denoted optimal for translation, it is necessary to specify what aspect of translation the codon is optimal for, and under what biological circumstances.

Many previous studies have attempted to provide explanations for why certain codons are used more frequently than others within a genome, or in particular genes. Here, we have examined related but distinct questions: why do closely related species use different codons, and use them preferentially at evolutionarily conserved sites in proteins? And how does this site-specific usage change across the developmental program? Our results do not conflict with the well-established influence of gene expression levels or tRNA abundances on codon usage bias (to choose two of several causal factors), but do indicate that existing models are incomplete in important ways. Our study provides molecular and mechanistic insights that must be incorporated into any large-scale integrated attempt to explain the evolution of codon usage within and between species.

Why were such clear, systematic, multi-species shifts in codon usage not found in previous analyses? Close examination of results in a previous study reveals that, using one analytical approach, virtually all of the same shifts we report are apparent, but were passed over in favor of other approaches to yield the conclusion that the preferred set of codons is quite constant across Drosophila [35] (cf. their Figure 2C). The approach in which results most closely match ours—analysis of relative synonymous codon usage (RSCU) in the top 10% most-biased genes as determined by their effective number of codons (ENC) [35]—has no particular mechanistic or evolutionary interpretation that differentiates it from similar approaches that gave different results. An analysis of codon usage in 69 ribosomal proteins across the 12 species reported a reversal of the most frequently used Asp codon, but not others, and argued that this change was minor and likely to be unimportant [36]. This result may reflect the restricted size or unusual constraints on the ribosomal protein-coding gene set; we argue that speed/accuracy conflicts may also explain the apparent differences between this analysis and ours. A systematic codon-usage shift in *D. willistoni* is well-documented [35],[47],[55], but this species appears in most analyses to be a strong outlier in its codings. Mutational biases appear to contribute to, but not fully explain, changes in codon usage in *willistoni* [36],[55], a conclusion our data support. A major advantage of the within-gene comparison introduced by Akashi, and exploited here, is that it controls for mutational biases that vary over large genomic regions and between chromosomes. That *willistoni* behaves much like related species in our analyses is consistent with the idea that mutational biases contribute to its outlier appearance, but not its codon-usage shift. Overall, it appears that previous studies have seen signs of the shifts we report, but without a mechanism-specific analytical approach, a strong control for confounding biases, and experimental knowledge of the tRNA modification, these signs failed to coalesce into a coherent picture.

Outside of the examples above, only one additional shift in codon usage has been identified in the drosophilids, a preference shift from UCC to AGC (serine) between *D. melanogaster* and *D. virilis*. This reflects small relative differences between three codons (including UCG), of six, that are all roughly equally preferred over their counterparts [35], such that the apparent change in preference is analogous to front-running athletes edging each other out rather than a fundamental change in the race. Why these changes have occurred remains unclear. Our study indicates that in terms of accuracy selection detectable using Akashi selection scores, serine codons remain quite stable, with a slight shift in the benefit of UCA relative to UCU. We identify several other accuracy-related shifts, most linked to changes in queuosine modification of tRNA, others (such as in isoleucine) less clearly so.

Our findings have implications for the recent discovery that selection on synonymous sites in the drosophilids is far stronger than previously appreciated [4]. This study concluded that standard explanations for selection on codons, such as translational speed and accuracy, could not account for this strong selection. To reach this conclusion, codons were designated optimal or non-optimal, and these assignments were assumed constant across the phylogeny (excepting *D. willistoni*) and over the course of development. The results here suggest all three assumptions overlook key features of codon usage in these animals: different codons can be optimal for different selective mechanisms, and the relative selective benefit of codons is not constant across the phylogeny nor across development. It may prove useful to revisit the causes of strong selective constraint on synonymous sites with a more nuanced model for how selection has acted on translation in the drosophilids.

The tRNA modification studied here, guanine to queuosine in the anticodon, has been studied for decades yet still has an unknown primary function. Given the many modifications targeting tRNA anticodons [30], we conjecture that this modification is only one of many which regulate the speed, fidelity, and possibly other aspects of translation in ways that leave evolutionary fingerprints. Our results expose multiple shifts in accuracy-driven codon usage coupled to changes in queuosine modification, many but not all of which our kinetic model can explain as consequences of the modification. We do not claim that all such shifts arise from Q modification; other factors may well contribute. However, other coordinated shifts in accuracy (such as those in isoleucine codons) may be linked to queuosine modification in ways we do not yet grasp. The parallel changes in relative accuracy of isoleucine codons between species and across development provide some evidence to suggest that our understanding of the effects of the tRNA modification is far from complete. We anticipate that further studies, population-genetic and biochemical, will deepen our understanding of the genomic upheavals exposed here.

What causes between-species and developmental variation in queuosine modification levels? Two forces are likely to be at work: regulation of Q modification by the TGT enzyme or upstream factors, and bioavailability of the precursor nutrient queuine. Several lines of evidence suggest that bioavailability provides the dominant selective force. If reduced Q modification is a regulatory effect, it should be largely independent of substrate availability. Contrary to this prediction, supplementation of free queuine to *D. melanogaster* third-instar larvae (the lowest-Q phase [38]) at nanomolar levels suffices to increase Q modification of tRNA several-fold [40]. Micromolar queuine supplementation leads to near-complete modification [40]. Free cellular queuine concentration is strongly positively correlated with tRNA modification level, a hallmark of a substrate-limited process [41]. Thus, substrate limitation, rather than regulation, appears to be the primary determinant of Q modification levels. Whether other species are substrate-limited for queuine like *D. melanogaster* remains an open question. Species-wide variation in Q modification may stem from differences in gut microbiota, consistent with the wide variation we observe in species reared on identical diets, or from host variation, such as differences in expression of the enzyme(s) responsible for liberating queuine for absorption.

Limiting queuine provides a simple explanation for the dip in Q modification during larval stages, and indeed the longstanding but poorly understood association between mitotic activity and reduced levels of queuosine tRNA modification, which is also observed in rapidly dividing cancer cells [56]. During rapid growth, such as larval development when mass (and thus tRNA content) increases more than 200-fold [40],[51], queuine intake must increase just to keep modification levels constant. If TGT is substrate-limited and the microbial sources of queuine multiply less rapidly than the growing organism, the exponential increases in tRNA abundance during rapid growth will result in transiently reduced Q modification, as observed in *D. melanogaster* [40].

Our results illuminate a surprising interplay between microbially acquired compounds, the fidelity of an organism's translational apparatus during development, and the evolutionary fate of its genome. Application of the general approaches introduced here to diverse taxa will likely yield more and deeper insights into this and similar novel modes of coevolutionary change.

## Materials and Methods

### Data Availability

Akashi selection scores for the 12 drosophilid species, and for genes maximally expressed at *D. melanogaster* developmental stages, may be accessed from the Dryad repository (datadryad.org) at doi:10.5061/dryad.1jn88 [57].

### Definition and Estimation of Akashi Selection Scores

Assuming weak selection, free recombination, and evolutionary steady-state, the log proportion of codon *I* relative to codon *J* is given by ln *p_I_^C^*/*p_J_^C^* = *M_IJ_*+*S_IJ_* where *M_IJ_* is the mutational bias (the log-ratio of mutation rates from *J* to *I*) and *S_IJ_* is the population-scaled additive selective (fitness) advantage of codon *J* over codon *I* (*S_IJ_* = *N_e_s_IJ_* with *N_e_* the effective population size) [58].

Let the proportion of sites with codon *I* that encode amino acids which are conserved across all 12 species be *p_I_^C^*, and at unconserved (variable) sites be *p_I_^V^*. At sites within the same protein, then,
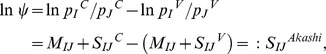
where *S_IJ_^Akashi^* is the Akashi selection score quantifying the population-scaled difference in selective advantage resulting from a change from reference codon *J* to codon *I* at a conserved site relative to that at a variable site in the same gene. This difference is attributable to translational accuracy. The left-hand side is the log-odds ratio for a 2×2 contingency table (conserved versus variable, codon *I* versus codon *J*) which can, given codon counts *n*, be estimated by *ψ* ˆ = ln *n_I_^C^*/*n_J_^C^*−ln *n_I_^V^*/*n_J_^V^*. Akashi pointed out that such log-odds ratios can be estimated using the Mantel-Haenszel procedure [59], which allows 2×2 tables to be computed for each gene separately and then combined into a single estimate, which, by construction, controls for all between-gene differences (such as levels of gene expression, structure, function, between-gene variation in mutational biases, and so on) which can distort other estimates of selection. With genes indexed by *g* and *n_g_* = *n_Ig_^C^*+*n_Jg_^V^+n_Ig_^V^+n_Jg_^C^*, the Mantel-Haenszel estimate is *ψ* ˆ = Σ*_g_* (*n_Ig_^C^ n_Jg_^V^/n_g_*)/Σ*_g_* (*n_Ig_^V^ n_Jg_^C^/n_g_*) with variance given by the Robins-Breslow-Greenland estimator [60]. These estimates are only approximately additive.

Selection coefficients quantify the fitness advantage of a genotype over a reference, and we take as a reference the lowest-relative-fitness codon in *D. melanogaster* in each synonymous family. That is, we choose the reference codon such that all synonymous changes from that codon are (in this analysis) beneficial in *D. melanogaster*.

We quantify Akashi selection scores for all possible pairs of synonyms (no score for single-codon families, one pair for two codons, three pairs for three codons, six pairs for four codons, and 15 pairs for six codons).

### Sequences, Alignments, and Gene Expression Data

Coding sequence alignments for 12 drosophilid species were obtained for 9,855 transcripts from FlyBase [61] (ftp://ftp.flybase.org/12_species_analysis/clark_eisen/alignments/all_species.guide_tree.cds.tar.gz), and filtered to include a single transcript per gene, aligned with 1∶1 orthologs in all 12 species (ftp://ftp.flybase.org/12_species_analysis/clark_eisen/homology/GeneWise.revised.homology.tsv.gz), with a minimum fraction alignable of 50% and at least 50 codons, yielding 5,182 alignments used for all analyses.

Maximal developmental-stage expression was evaluated using published data [51], which, among the alignments above, yielded 1055, 675, 885, 502, 891, and 301 genes with maximal expression in early embryo (E0), late embryo (E3), larva (L), pupa (P), adult male (M), and adult female (F) flies, respectively. Embryo (E) genes were those with maximal expression in either E0 or E3, and adult (A) genes were those with maximal expression in M or F.

### Tissue Collection and RNA Extraction

All drosophilid species were reared in bottles on standard yeast-glucose media at room temperature (approximately 23°C). RNA was extracted from third instar larvae and 2-week-old adults using the standard TRIzol (Invitrogen) protocol. For larval collection, adults were placed on fresh food for 24 hours, after which great care was used to ensure that all flies were removed. We noted when larvae first started the roaming stage. 24 hours later larvae were collected from the bottles by pouring enough 5 M NaCl to cover the media and allowing the resulting mixture to set for 5 minutes. Floating larvae were poured onto mesh and washed in water before snap-freezing in liquid nitrogen. To age adults, newly eclosed flies were transferred to fresh bottles and every few days transferred to new bottles. After two weeks the flies were snap-frozen in liquid nitrogen.

### Detection of Q Modification using Acryloyl Aminophenylboronic Acid Gel

This method was based on the protocol developed by Igloi and Kössel [48]. 2.5 µg of total RNA was deacylated by incubating in 100 mM TrisHCl (pH 9) for 30 min at 37°C. The deacylated RNA was combined with an equal volume of denaturing gel loading buffer containing 8 M urea, 5% glycerol, 0.05% bromophenol blue, and 0.05% xylene cyanol. Samples were loaded onto denaturing 10% polyacrylamide gels containing 5% 3-aminophenylboronic acid (Boron Molecular) and gel electrophoresis was run at 4°C in TAE. RNA was transferred under vacuum by layering 3MW blotting paper (MIDSCI), Hybond-XL membrane (GE Healthcare), gel, and plastic wrap on a gel dryer for 2 h at 80°C. After transfer the gel was removed from the membrane by soaking in distilled water. The membrane was washed twice for 30 min each in hybridization buffer (20 mM phosphate, pH 7, 300 mM NaCl, 1% SDS), followed by incubation with 5′ ^32^P-labeled DNA oligonucleotide probes in the hybridization buffer for 16 h at 60°C. Membranes were washed three times for 20 min each in a solution containing 20 mM phosphate (pH 7.2), 300 mM NaCl, 2 mM EDTA, and 0.1% SDS and exposed to phosphor-imaging plates. Band intensity was quantified using software from the PhosphorImager manufacturer (Fuji Medicals).

### Periodate Oxidation Control

Total RNA was first deacylated as described above. The deacylated RNA was incubated in 100 mM NaOAc/HOAc (pH 4.5) and 50 mM freshly prepared periodate (NaIO_4_) at room temperature for 30 min. 100 mM glucose was added and the mixture incubated for another 5 min. The mixture was run through a pre-equilibrated G25 column (GE Healthcare) to remove periodate followed by ethanol precipitation. Sample was then dissolved in the denaturing gel loading buffer.

### Northern Blot of mRNA

7 µg of total RNA was incubated at 55°C for 15 min in 7% formaldehyde, 50% formamide, and 0.5× running buffer (1× running buffer is 200 mM MOPS, pH 7, 80 mM NaOAc, 10 mM EDTA). Samples were then combined with an equal volume of gel loading buffer (5% glycerol, 0.05% bromophenol blue, and 0.05% xylene cyanol), and loaded onto 0.8% agarose gels (0.8% agarose, 1× running buffer, 2% formaldehyde). After electrophoresis, the gel was washed for 15 min in distilled water and 15 min in 10× SSC. RNA was transferred by capillary blotting overnight. After transfer the RNA was cross-linked to the membrane at 70,000 µJ/cm^2^. The membrane was washed, hybridized, and exposed in the same manner as described for the acryloyl aminophenylboronic acid gel shift assay.

Oligonucleotide probe sequences were: tRNA^His^:

5′-TGCCGTGACCAGGATTCGAACCTGGGTTACCACGGCCACAACGTGGGGTCCTAACCACTAGACGATCACGGC;

tRNA^Tyr^:5′-TCCTTCGAGCCGGASTCGAACCAGCGACCTAAGGATCTACAGTCCTCCGCTCTACCARCTGAGCTATCGAAGG;

tRNA^Asn^:5′-CGTCCCTGGGTGGGCTCGAACCACCAACCTTTCGGTTAACAGCCGAACGCGCTAACCGATTGCGCCACAGAGAC;

TGT mRNA:5′-CGATCCACCCARCGWATDGTVCGCTCCATRGCCTC;

Actin mRNA:5′-CTTCTCCTTGATGTCRCGNACRATTTCACGCTCAGCSGTGGTGGTGAA

### Kinetic Model for a Shift in Codon Usage

We observe that the proportion of Q-tRNA rises (and G-tRNA correspondingly decreases) as C-ending codons rise in inferred accuracy relative U-ending codons. To understand this shift, we assume that (1) relevant tRNAs read C-ending codons more rapidly than U-ending codons; (2) a competitor tRNA bearing another amino acid, such that mistranslation would occur if this tRNA were accepted, also reads C-ending codons more rapidly than U-ending codons; (3) ribosomes translate cognate codons using Q-tRNA more rapidly than using G-tRNA.

We adapt a previously introduced framework to build a kinetic model [52]. Let the first-order rate constant of X-tRNA for reading NAY (Y = C or U) codons be *k_XY_* and the concentration of X-tRNA be [*X*]. Then, for example, the rate of translation of NAC codons by Q-tRNA is *k_QC_*[*Q*]. For simplicity, we model competitor tRNAs using a single “effective” tRNA with concentration [*M*] and reading rate constants k_M*_. The rate of translation of Y-ending codons is *r_Y_* = *k_QY_*[*Q*]+*k_GY_*[*G*]+*k_MY_*[*M*], and the proportion of mistranslated Y-ending codons is *ϵ_Y_* = *k_MY_*[*M*]/*r_Y_*. We denote the proportion of Q-modified tRNA as *q* = [*Q*]/[*T*] with total cognate tRNA concentration [*T*] = [*Q*]+[*G*], and further assume that the competitor tRNA is present at a concentration *α* times that of the cognate tRNA, [*M*] = α[*T*]. In our simulations, we choose α = 1 for simplicity. Then the error rate of a Y-ending codon (Y = C or U), as a function of the proportion of Q-modified tRNA, is *ϵ_Y_*(*q*) = *k_MY_*α/[*k_MY_*α+*k_QY_q+k_GY_*(1−*q*)]. R source code to reproduce the graphs in Figure 5 is included as Listing S1.

## Supporting Information

Figure S1 **tRNA-guanine transglycosylase (TGT) gene expression.** Northern blotting with probes to TGT and actin (top), with quantification (bottom; error bars show standard error of the mean [SEM]).
(TIFF)

Table S1 **Top-scoring codons in diverse analyses of** ***D. melanogaster*** **broadly agree.** Shaded cells indicate cases where one analysis produces a uniquely different top-scoring codon.
(DOCX)

Table S2 **Akashi selection scores across 12 drosophilid species.**
(DOCX)

Table S3 **tRNA gene counts for 12 drosophilid species.**
(DOCX)

Data S1 **Quantification of queuosine modification (from** **Figure 3** **) and TGT expression levels (Figure S1).**
(XLSX)

Listing S1 **R script of the kinetic model used to generate** **Figure 5** **.**
(DOCX)
