# Correction: A Nutrient-Driven tRNA Modification Alters Translational Fidelity and Genome-wide Protein Coding across an Animal Genus

**DOI:** 10.1371/journal.pbio.1002150

**Published:** 2015-04-29

**Authors:** John M. Zaborske, Vanessa L. Bauer DuMont, Edward W. J. Wallace, Tao Pan, Charles F. Aquadro, D. Allan Drummond

Since publication of this paper, the authors became aware of some incorrect or missing details that require rectification. The authors have provided corrected versions, along with explanations, here.

1. The original Fig 2 is missing a double bond in the three cyclopentyl moieties. A corrected Fig 2 is provided here. This new Fig 2 also adds stereochemistry to that moiety.
2. The authors identified some errors within the manuscript text in referencing Fig 5 (unaltered original figure is provided here for reference). These have now been corrected in the paragraph below. A corrected version of the manuscript file is also provided here (see S1 Manuscript below).

**Fig 2:**
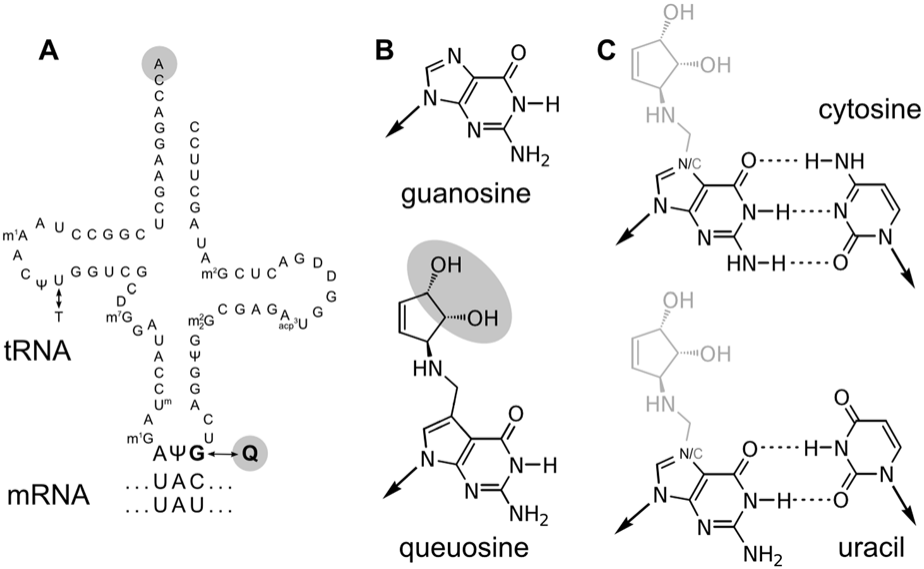
Queuosine modification alters features of tRNA anticodons. (A) The sequence of D. melanogaster tRNATyr (after Suter and colleagues [62]) shows modification of guanosine (G) to queuosine (Q) in the anticodon position corresponding to the third-position (wobble) base of the codon. Positions of cis-diol moieties are highlighted in gray. (B) Guanosine (top) and queuosine (bottom); cis-diol highlighted in gray. Arrows point toward the primary ribose moiety which is not shown. (C) Guanosine and queuosine binding cytosine (C, top) and uracil (U, bottom).

**Fig 5:**
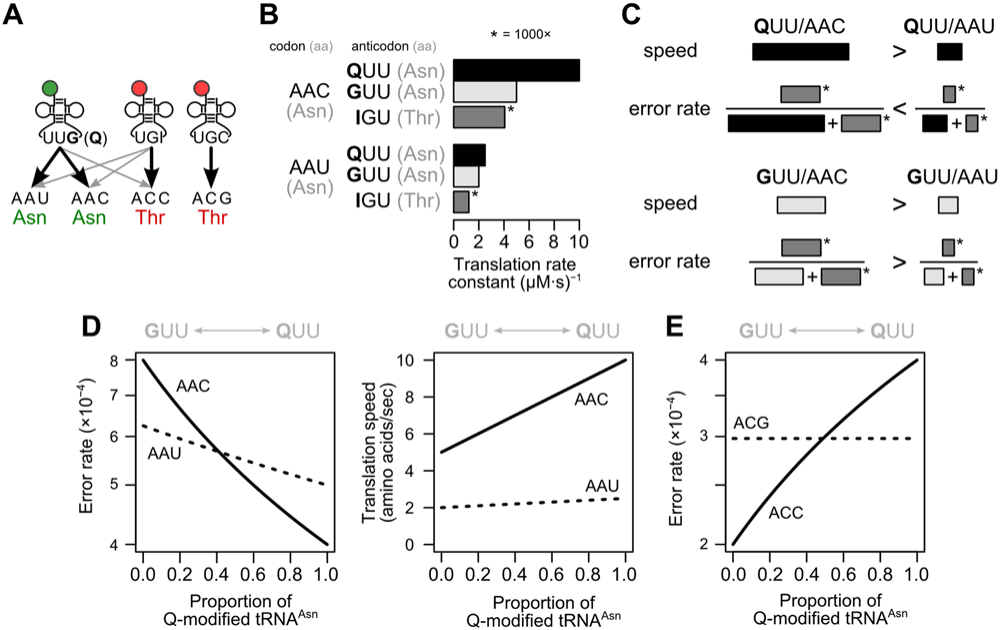
A kinetic competition model illustrates how Q modification alone can reverse relative codon accuracy. (A) Schematic representation of tRNA and codon relationships. Black lines represent cognate tRNA/codon relationships, and gray lines represent non-cognate (misreading) relationships. (B) A kinetic model produces rates for each tRNA reading the two asparagine codons AAC and AAU (cf. Listing S1). Misreading rates by tRNAThr(IGU) are multiplied by 1,000 for visibility. The translation rate constant is proportional to the translation rate assuming equal tRNA concentrations, which we do for simplicity. (C) Graphical view of how rates given in (B) combine to produce speeds and error rates for each codon/tRNA pair. In the example, tRNAAsn(QUU) reads AAC faster and more accurately. In contrast, tRNAAsn(GUU) reads AAC faster, but AAU more accurately. (D) Quantitative error rates and translation speeds as a function of Q-modification in the model. (E) Modeled accuracy of the threonine codon ACC, which is assumed to be misread by tRNAAsn, changes with Q-modification, whereas ACG, which is not misread by tRNAAsn, does not, again resulting in a shift in relative accuracy.

The paragraphs containing corrected references to Fig 5 (explained in item 2 above) can be found below. In the original manuscript PDF, this section of text begins at the last paragraph on page 7:

The model assumes that G-tRNA, Q-tRNA, and the near-cognate I-tRNA all have higher first-order rate constants for reading C-ending codons than for U-ending codons (Fig 5A–5C), consistent with *in vitro* binding studies [31]. Q-tRNA is assumed to bind more rapidly than G-tRNA to any given codon, consistent with a higher affinity of Q-modified tRNA for ribosomes [45]. Finally, the relative rate of Q-tRNA binding C-ending over U-ending codons is assumed higher than for G-tRNA. Under these assumptions, the identity of the most accurately translated (lowest error rate) synonymous codon in a family can switch from C-ending to U-ending solely as a function of changes in queuosine modification (Fig 5D). This model generates error rates (between 10^-4^ and 10^-3^) and translation speeds (1–10 amino acids per second) matching physiological estimates [53,54] (Fig 5D). While the model’s precise parameters are surely inaccurate, its value lies in showing that tRNA modification alone is capable of inducing an accuracy reversal under biologically plausible conditions.

The kinetic competition model offers a unique and intuitive explanation for why codons that are not normally read by a Q-modified tRNA nonetheless shift in accuracy when Q modification levels change: these codons are misread by Q-tRNA. If Q modification primarily increases tRNA affinity for ribosomes, then increased Q modification will reduce the accuracy of near-cognate codons due to misreading by Q-tRNA (Fig 5E). This accuracy reduction is detectable as a reduced Akashi selection score. Kinetically, accuracy reduction arises when we consider the inverse of the above problem: misreading of threonine ACC codons by G/Q-tRNA^Asn^ (which would properly read AAC/AAU codons). Given the apparent Q-modified tRNA, we can predict that ACC and ACU will be misread more often by Q-tRNA^Asn^ than will ACG, which is read by a separate tRNA, tRNA^Thr^(CGU). Consistent with this prediction, ACC is deleterious relative to ACG in the *melanogaster* subgroup where Q modification is highest, and beneficial in *D*. *virilis* where Q modification is nearly absent (Fig 1A and Table S2). Indeed, the relative benefits of A- or G-ending codons compared to U- or C-ending synonym change similarly for six amino acids (Gly, Thr, Val, Pro, Ser and Leu) (Fig 1A and Table S2).

Most but not all observed codon-usage shifts can be explained by this kinetic model. The major exception is isoleucine, for which the A-ending codon AUA has an accuracy benefit over AUC/AUU in *virilis* but a cost in *melanogaster*. The isoleucine codon AUA is costly relative to AUU in every developmental stage except for larva, the lowest-Q stage, where the fitness cost becomes insignificant. This change mirrors the changes in accuracy benefit of these two codons in *D*. *virilis*, the lowest-Q species in our measurements, suggesting a link to the modification which is not captured by our kinetic model.

The kinetic model predicts that, unlike accuracy, the relative speed of codons always favors C-ending codons regardless of the level of Q modification (Fig 5D). That is, speed and accuracy selection can come into conflict dependent on the modification level, where one codon is more accurately translated but less rapidly translated than its synonym. If selection for speed were strong enough for a set of genes, those genes would show little or no accuracy-driven shift. A previous analysis found that genes encoding ribosomal proteins show consistent use of C-ending codons for His/Asn/Tyr across the phylogeny, but Asp codons shift in usage from C-ending to U-ending [18]. Because selection on speed favors increased production of ribosomes, ribosomal proteins may be expected to bear strong signatures of speed selection in addition to accuracy selection, making them unusually subject to speed/accuracy conflicts. We hypothesize that in most cases the speed benefit overwhelms the accuracy cost of C-ending codons in low-Q conditions for His/Asn/Tyr, but that accuracy costs outweigh speed benefits for Asp—perhaps because Asp codon mistranslation yields products that are particularly disruptive to ribosomal assembly or function. Our hypothesis illustrates the larger principle that the outcome of speed/accuracy conflicts can be amino-acid-specific, depending upon the consequences of speed and accuracy differences for each synonymous codon.

## Supporting Information

S1 Manuscript Corrected manuscript file. (DOCX)
