# Supplementary figures and images for: A Nutrient-Driven tRNA Modification Alters Translational Fidelity and Genome-wide Protein Coding across an Animal Genus
Source: PLoS Biol. 2014 Dec 9;12(12):e1002015. doi: 10.1371/journal.pbio.1002015 (PMC4260829; doi:10.1371/journal.pbio.1002015)

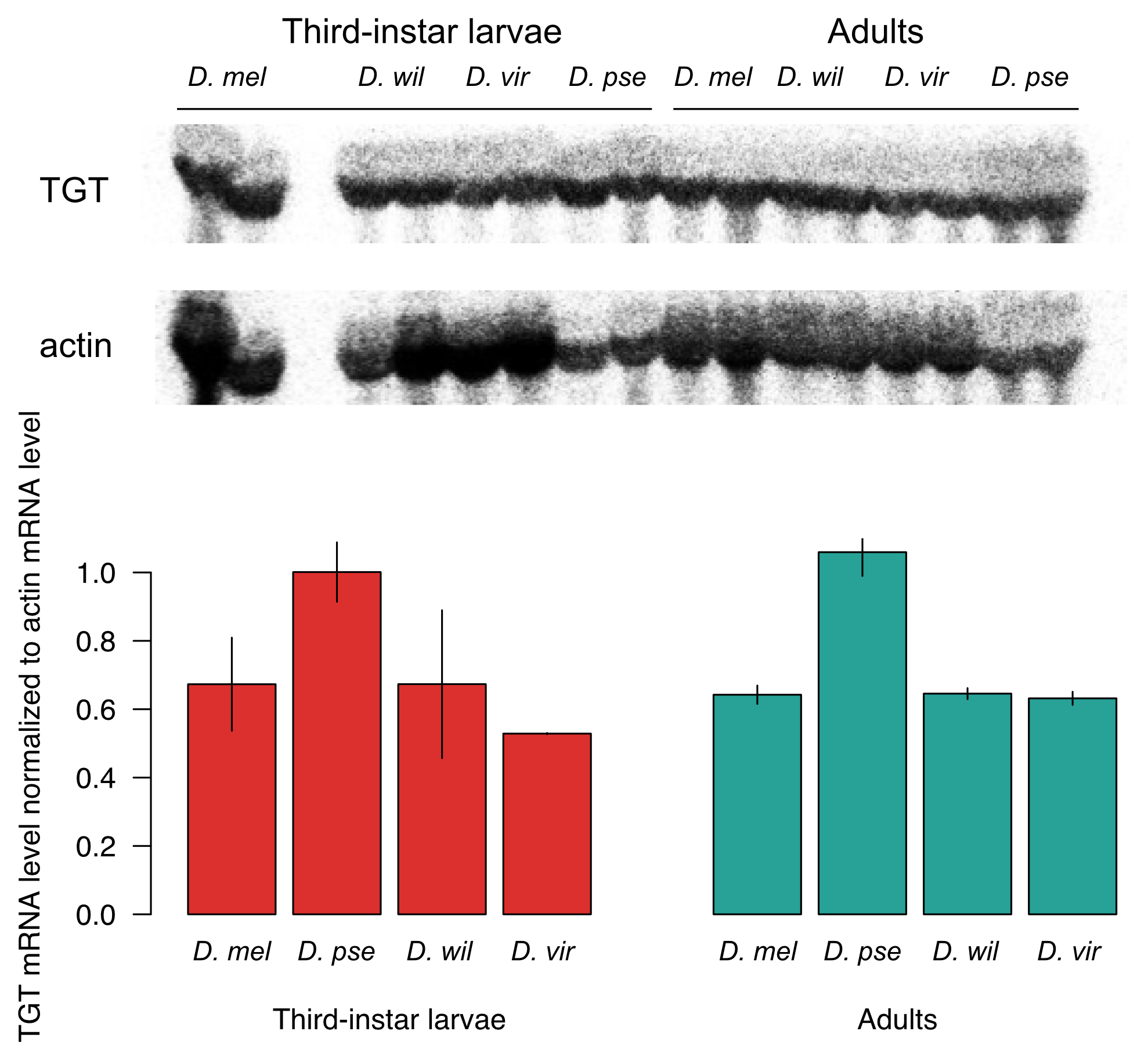

Supplement: Figure S1 — tRNA-guanine transglycosylase (TGT) gene expression. Northern blotting with probes to TGT and actin (top), with quantification (bottom; error bars show standard error of the mean [SEM]). (TIFF) [file pbio.1002015.s001.tiff]
