# Supplementary material for: A Nutrient-Driven tRNA Modification Alters Translational Fidelity and Genome-wide Protein Coding across an Animal Genus
Source: PLoS Biol. 2014 Dec 9;12(12):e1002015. doi: 10.1371/journal.pbio.1002015 (PMC4260829; doi:10.1371/journal.pbio.1002015)
Supplement: Table S1 — Top-scoring codons in diverse analyses of D. melanogaster broadly agree. Shaded cells indicate cases where one analysis produces a uniquely different top-scoring codon. (DOCX) [file pbio.1002015.s002.docx]

**Table S1**: Top-scoring codons in diverse analyses show broad agreement in *D. melanogaster*. Shaded cells indicate cases where one analysis produces a uniquely different top-scoring codon.

| **Amino acid** | **“Favored” codon(s) [1]** | **“Optimal” codon [2]** | **Highest frequency in ribosomal protein genes [3]** | **Most beneficial Akashi selection score  (this study)** |
| --- | --- | --- | --- | --- |
| A | GCC | GCC | GCC | GCC |
| C | UGC | UGC | UGC | UGC |
| D | GAC | GAC | GAC | GAC |
| E | GAG | GAG | GAG | GAG |
| F | UUC | UUC | UUC | UUC |
| G | GGC | GGC | GGC | GGC |
| H | CAC | CAC | CAC | CAC |
| I | AUC | AUC | AUC | AUC |
| K | AAG | AAG | AAG | AAG |
| L | CUC/CUG | CUG | CUG | CUG |
| N | AAC | AAC | AAC | AAC |
| P | CCC | CCC | CCC | CCC |
| Q | CAG | CAG | CAG | CAG |
| R | CGC/CGU | CGC | CGG | CGC |
| S | UCC/UCG | AGC | UCC | UCG |
| T | ACC | ACC | ACC | ACG |
| V | GUC/GUG | GUC | GUG | GUG |
| Y | UAC | UAC | UAC | UAC |

1. Duret L, Mouchiroud D (1999) Expression pattern and, surprisingly, gene length shape codon usage in Caenorhabditis, Drosophila, and Arabidopsis. Proc Natl Acad Sci U S A 96: 4482-4487.

2. Zhou T, Weems M, Wilke CO (2009) Translationally optimal codons associate with structurally sensitive sites in proteins. Mol Biol Evol 26: 1571-1580.

3. Heger A, Ponting CP (2007) Variable strength of translational selection among 12 Drosophila species. Genetics 177: 1337-1348.
