# Supplementary material for: A Nutrient-Driven tRNA Modification Alters Translational Fidelity and Genome-wide Protein Coding across an Animal Genus
Source: PLoS Biol. 2014 Dec 9;12(12):e1002015. doi: 10.1371/journal.pbio.1002015 (PMC4260829; doi:10.1371/journal.pbio.1002015)
Supplement: Table S2 — Akashi selection scores across 12 drosophilid species. (DOCX) [file pbio.1002015.s003.docx]

**Table S2**: Akashi selection scores for 12 drosophilid species.

| aa | reference codon | codon | dmel | dsim | dsec | dere | dyak | dana | dpse | dper | dwil | dgri | dmoj | dvir |
| --- | --- | --- | --- | --- | --- | --- | --- | --- | --- | --- | --- | --- | --- | --- |
| A | GCA | GCC | 0.3257 | 0.3368 | 0.3474 | 0.3394 | 0.3455 | 0.3808 | 0.4682 | 0.4898 | 0.3947 | 0.337 | 0.3385 | 0.3086 |
| A | GCA | GCG | 0.0423 | 0.0469 | 0.0501 | 0.018 | 0.0375 | 0.1054 | 0.1191 | 0.1233 | 0.0237 | 0.1571 | 0.1575 | 0.1717 |
| A | GCA | GCT | 0.1674 | 0.1699 | 0.1696 | 0.1748 | 0.1802 | 0.2209 | 0.219 | 0.2141 | 0.2512 | 0.0547 | 0.1068 | 0.0516 |
| A | GCT | GCC | 0.162 | 0.1691 | 0.1845 | 0.1696 | 0.1586 | 0.1557 | 0.2336 | 0.2518 | 0.1362 | 0.2723 | 0.2167 | 0.2477 |
| A | GCG | GCT | 0.1253 | 0.1276 | 0.1158 | 0.1526 | 0.1445 | 0.0978 | 0.1114 | 0.1093 | 0.222 | -0.0923 | -0.061 | -0.1133 |
| A | GCG | GCC | 0.2968 | 0.3031 | 0.3093 | 0.3204 | 0.309 | 0.2671 | 0.3528 | 0.3648 | 0.3724 | 0.1777 | 0.1757 | 0.1371 |
| C | TGT | TGC | 0.1291 | 0.135 | 0.0945 | 0.0614 | 0.0869 | -0.032 | 0.0395 | 0.0988 | -0.0097 | 0.0082 | -0.025 | 0.0021 |
| D | GAT | GAC | 0.1031 | 0.1157 | 0.1119 | 0.078 | 0.079 | 0.0796 | -0.006 | -0.0033 | -0.163 | -0.1388 | -0.1011 | -0.1145 |
| E | GAA | GAG | 0.2329 | 0.2269 | 0.2365 | 0.2343 | 0.2385 | 0.2081 | 0.3068 | 0.3162 | 0.1149 | 0.1604 | 0.1882 | 0.1744 |
| F | TTT | TTC | 0.0789 | 0.0964 | 0.0792 | 0.0821 | 0.0717 | 0.0968 | 0.0669 | 0.074 | 0.0948 | 0.0531 | 0.0795 | 0.0335 |
| G | GGG | GGA | 0.1731 | 0.192 | 0.1637 | 0.2206 | 0.2317 | 0.1229 | 0.1523 | 0.1294 | 0.1658 | 0.2593 | 0.2217 | 0.1711 |
| G | GGG | GGC | 0.2525 | 0.2898 | 0.2543 | 0.2978 | 0.2977 | 0.2131 | 0.3106 | 0.3143 | 0.3431 | 0.3976 | 0.3285 | 0.3136 |
| G | GGG | GGT | 0.1377 | 0.1521 | 0.1348 | 0.1896 | 0.2124 | 0.1476 | 0.143 | 0.1299 | 0.3559 | 0.3529 | 0.2613 | 0.2391 |
| G | GGA | GGC | 0.0786 | 0.09 | 0.0907 | 0.0589 | 0.0621 | 0.0858 | 0.1378 | 0.1657 | 0.183 | 0.1282 | 0.1225 | 0.1137 |
| G | GGT | GGA | 0.0043 | 0.0049 | 0.0042 | 0.0541 | 0.0407 | -0.0242 | 0.0086 | -0.0101 | -0.1921 | -0.0846 | -0.056 | -0.0411 |
| G | GGT | GGC | 0.0955 | 0.1023 | 0.0995 | 0.1168 | 0.0933 | 0.052 | 0.1434 | 0.1558 | -0.0149 | 0.0381 | 0.0797 | 0.0401 |
| H | CAT | CAC | 0.0768 | 0.08 | 0.0856 | 0.0789 | 0.0728 | 0.1199 | -0.0003 | 0.0075 | -0.0966 | -0.0558 | -0.019 | -0.0693 |
| I | ATA | ATC | 0.1712 | 0.1754 | 0.1736 | 0.1717 | 0.171 | 0.1941 | 0.0481 | 0.0569 | 0.0316 | -0.1551 | -0.069 | -0.1402 |
| I | ATA | ATT | 0.1081 | 0.1055 | 0.1119 | 0.1098 | 0.1235 | 0.1681 | 0.0778 | 0.0904 | 0.1083 | -0.0393 | 0.0095 | -0.0184 |
| I | ATT | ATC | 0.0576 | 0.0651 | 0.0552 | 0.0644 | 0.0537 | 0.034 | -0.0294 | -0.0298 | -0.0741 | -0.1084 | -0.0674 | -0.1147 |
| K | AAA | AAG | 0.1652 | 0.1776 | 0.1671 | 0.1594 | 0.1598 | 0.1347 | 0.182 | 0.1834 | 0.0254 | 0.1109 | 0.1213 | 0.0956 |
| L | CTT | CTA | 0.0744 | 0.0602 | 0.055 | 0.0426 | 0.0655 | 0.0594 | 0.0901 | 0.1173 | 0.0715 | 0.2379 | 0.145 | 0.1575 |
| L | CTT | CTC | 0.1139 | 0.1206 | 0.1192 | 0.0983 | 0.1031 | 0.1328 | 0.1895 | 0.2208 | 0.1219 | 0.3189 | 0.2605 | 0.2389 |
| L | CTT | CTG | 0.1713 | 0.1967 | 0.1849 | 0.1589 | 0.1505 | 0.1678 | 0.2721 | 0.3051 | 0.1158 | 0.3206 | 0.2404 | 0.2319 |
| L | CTA | CTC | 0.0267 | 0.0419 | 0.0523 | 0.0562 | 0.0412 | 0.0972 | 0.1081 | 0.1148 | 0.0444 | 0.0963 | 0.1334 | 0.0894 |
| L | CTA | CTG | 0.0823 | 0.1108 | 0.1103 | 0.0998 | 0.0816 | 0.1288 | 0.169 | 0.1793 | 0.0423 | 0.0835 | 0.0983 | 0.062 |
| L | CTC | CTG | 0.0579 | 0.0785 | 0.0791 | 0.0564 | 0.0494 | 0.0291 | 0.0559 | 0.0623 | -0.0023 | -0.022 | -0.0222 | -0.0357 |
| L | TTA | TTG | 0.1731 | 0.1549 | 0.1941 | 0.1281 | 0.0717 | 0.1969 | 0.2749 | 0.2784 | 0.152 | 0.1964 | 0.1311 | 0.175 |
| N | AAT | AAC | 0.0347 | 0.0325 | 0.0327 | 0.0621 | 0.0509 | 0.0447 | -0.0387 | -0.0363 | -0.2397 | -0.206 | -0.1855 | -0.198 |
| P | CCT | CCA | 0.0457 | 0.0374 | 0.0305 | 0.0035 | 0.0067 | -0.041 | 0.0149 | 0.0308 | -0.0076 | 0.044 | -0.0276 | -0.0616 |
| P | CCT | CCC | 0.2854 | 0.2846 | 0.2707 | 0.2675 | 0.2941 | 0.2041 | 0.3933 | 0.4271 | 0.2694 | 0.3895 | 0.2907 | 0.2712 |
| P | CCT | CCG | 0.1604 | 0.1672 | 0.1515 | 0.1424 | 0.147 | 0.115 | 0.2407 | 0.2694 | 0.1006 | 0.242 | 0.159 | 0.1 |
| P | CCA | CCC | 0.2451 | 0.2654 | 0.2617 | 0.2634 | 0.267 | 0.2526 | 0.3837 | 0.3984 | 0.2752 | 0.3381 | 0.3276 | 0.3497 |
| P | CCA | CCG | 0.1243 | 0.1638 | 0.1509 | 0.1298 | 0.1402 | 0.1704 | 0.2374 | 0.2549 | 0.1036 | 0.2013 | 0.1789 | 0.1804 |
| P | CCG | CCC | 0.1194 | 0.1125 | 0.1153 | 0.1285 | 0.1226 | 0.0839 | 0.1463 | 0.1465 | 0.1761 | 0.1557 | 0.1586 | 0.1778 |
| Q | CAA | CAG | 0.1211 | 0.1335 | 0.1391 | 0.1264 | 0.1216 | 0.1293 | 0.1609 | 0.1977 | 0.0386 | 0.1264 | 0.07 | 0.0949 |
| R | AGG | AGA | 0.1692 | 0.1482 | 0.1316 | 0.1485 | 0.137 | 0.0712 | 0.1415 | 0.1441 | 0.0836 | 0.2067 | 0.2123 | 0.2498 |
| R | CGG | CGA | 0.1549 | 0.1459 | 0.123 | 0.1682 | 0.1395 | 0.1091 | 0.1086 | 0.0864 | 0.059 | 0.1252 | 0.0371 | 0.0997 |
| R | CGG | CGC | 0.3552 | 0.3656 | 0.3582 | 0.3657 | 0.3577 | 0.3042 | 0.4486 | 0.4655 | 0.3533 | 0.445 | 0.3689 | 0.4499 |
| R | CGG | CGT | 0.2877 | 0.2763 | 0.2619 | 0.3028 | 0.3073 | 0.2793 | 0.3628 | 0.3738 | 0.3171 | 0.3795 | 0.2834 | 0.3597 |
| R | CGA | CGC | 0.1973 | 0.2217 | 0.2354 | 0.2015 | 0.2003 | 0.2089 | 0.3498 | 0.3804 | 0.2686 | 0.3516 | 0.3374 | 0.3359 |
| R | CGA | CGT | 0.125 | 0.119 | 0.1275 | 0.1394 | 0.1606 | 0.1587 | 0.2674 | 0.2872 | 0.2454 | 0.2709 | 0.2627 | 0.2382 |
| R | CGT | CGC | 0.0782 | 0.1001 | 0.1115 | 0.0825 | 0.0548 | 0.0277 | 0.0758 | 0.0785 | 0.0286 | 0.0685 | 0.0654 | 0.0838 |
| S | AGC | AGT | 0.0076 | 0.0267 | 0.0227 | 0.0252 | 0.0332 | 0.0102 | 0.0507 | 0.0448 | 0.116 | 0.1083 | 0.0627 | 0.1223 |
| S | TCT | TCA | 0.0474 | 0.0252 | 0.03 | 0.0151 | 0.0438 | 0.005 | -0.0312 | -0.0119 | 0.0557 | 0.0196 | -0.0412 | -0.0075 |
| S | TCT | TCC | 0.1756 | 0.2103 | 0.2172 | 0.2095 | 0.2201 | 0.1814 | 0.2132 | 0.2485 | 0.215 | 0.2558 | 0.1733 | 0.1781 |
| S | TCT | TCG | 0.2135 | 0.242 | 0.2548 | 0.2447 | 0.247 | 0.2544 | 0.3289 | 0.3677 | 0.2778 | 0.2993 | 0.187 | 0.2441 |
| S | TCA | TCC | 0.1469 | 0.1839 | 0.1866 | 0.1655 | 0.1738 | 0.1849 | 0.2321 | 0.2518 | 0.1724 | 0.2726 | 0.2541 | 0.1976 |
| S | TCA | TCG | 0.1822 | 0.2235 | 0.2338 | 0.2063 | 0.2094 | 0.2506 | 0.3399 | 0.3644 | 0.2361 | 0.2946 | 0.2417 | 0.2271 |
| S | TCC | TCG | 0.0317 | 0.0322 | 0.0341 | 0.0371 | 0.0293 | 0.0914 | 0.1035 | 0.1102 | 0.0594 | 0.037 | -0.0095 | 0.0439 |
| T | ACT | ACA | 0.0776 | 0.0854 | 0.1037 | 0.0896 | 0.0675 | 0.0079 | 0.0576 | 0.0635 | 0.0051 | 0.1464 | 0.1401 | 0.0641 |
| T | ACT | ACC | 0.1445 | 0.1795 | 0.1789 | 0.1605 | 0.166 | 0.0969 | 0.189 | 0.2085 | 0.1396 | 0.3209 | 0.2749 | 0.289 |
| T | ACT | ACG | 0.1564 | 0.1798 | 0.1783 | 0.1594 | 0.1612 | 0.1334 | 0.1908 | 0.2036 | -0.008 | 0.2799 | 0.2571 | 0.2109 |
| T | ACA | ACC | 0.0797 | 0.0953 | 0.0803 | 0.081 | 0.1011 | 0.0985 | 0.1347 | 0.1433 | 0.134 | 0.1806 | 0.1457 | 0.2165 |
| T | ACA | ACG | 0.0888 | 0.1004 | 0.0792 | 0.1038 | 0.1028 | 0.136 | 0.1587 | 0.1589 | -0.0103 | 0.1257 | 0.1193 | 0.1451 |
| T | ACC | ACG | 0.0067 | 0.0023 | 0.006 | 0.0099 | 0.0022 | 0.0242 | 0.017 | 0.0091 | -0.159 | -0.0687 | -0.0493 | -0.109 |
| V | GTT | GTA | 0.0761 | 0.0663 | 0.07 | 0.0527 | 0.073 | 0.0013 | -0.0309 | -0.0294 | 0.0229 | -0.0977 | -0.0085 | 0.094 |
| V | GTT | GTC | 0.0467 | 0.0596 | 0.052 | 0.0435 | 0.0601 | -0.0015 | 0.0999 | 0.1203 | 0.0645 | 0.1534 | 0.1268 | 0.1441 |
| V | GTT | GTG | 0.266 | 0.294 | 0.2866 | 0.2857 | 0.3005 | 0.1994 | 0.2906 | 0.3057 | 0.183 | 0.2766 | 0.3108 | 0.3374 |
| V | GTC | GTA | 0.0249 | 0.0086 | 0.0239 | 0.0235 | 0.0049 | 0.0058 | -0.1343 | -0.1431 | -0.0563 | -0.2241 | -0.1212 | -0.0335 |
| V | GTA | GTG | 0.2118 | 0.2456 | 0.2373 | 0.2398 | 0.2506 | 0.1957 | 0.3303 | 0.3408 | 0.1615 | 0.3563 | 0.3049 | 0.2471 |
| V | GTC | GTG | 0.2265 | 0.2322 | 0.2404 | 0.2453 | 0.2408 | 0.1894 | 0.2056 | 0.2122 | 0.1093 | 0.1097 | 0.1631 | 0.1848 |
| Y | TAT | TAC | 0.017 | 0.0188 | 0.0087 | 0.0463 | 0.0523 | 0.1092 | 0.0301 | 0.0381 | -0.0911 | -0.0522 | -0.0281 | -0.0199 |
