# Supplementary material for: A Nutrient-Driven tRNA Modification Alters Translational Fidelity and Genome-wide Protein Coding across an Animal Genus
Source: PLoS Biol. 2014 Dec 9;12(12):e1002015. doi: 10.1371/journal.pbio.1002015 (PMC4260829; doi:10.1371/journal.pbio.1002015)
Supplement: Table S3 — tRNA gene counts for 12 drosophilid species. (DOCX) [file pbio.1002015.s004.docx]

**Table S3**: tRNA gene counts for 12 drosophilid species [1,2].

| aa | anticodon  3’-5’ | D.mel | D.sim | D.sec | D.ere | D.yak | D.ana | D.pse | D.per | D.wil | D.gri | D.moj | D.vir |
| --- | --- | --- | --- | --- | --- | --- | --- | --- | --- | --- | --- | --- | --- |
| A | CGA | 12 | 9 | 14 | 12 | 20 | 10 | 12 | 12 | 11 | 8 | 9 | 9 |
|  | CGC | 3 | 5 | 3 | 3 | 5 | 6 | 3 | 3 | 2 | 3 | 3 | 3 |
|  | CGU | 2 | 0 | 2 | 2 | 2 | 4 | 5 | 3 | 6 | 3 | 4 | 3 |
| C | ACA | 0 | 0 | 0 | 1 | 0 | 1 | 0 | 0 | 0 | 0 | 0 | 0 |
|  | ACG | 7 | 7 | 6 | 6 | 8 | 6 | 10 | 8 | 12 | 6 | 8 | 8 |
| D | CUG | 14 | 9 | 14 | 14 | 15 | 13 | 18 | 18 | 16 | 12 | 12 | 13 |
| E | CUC | 14 | 10 | 13 | 12 | 14 | 13 | 17 | 14 | 12 | 10 | 11 | 10 |
|  | CUU | 6 | 5 | 5 | 6 | 6 | 6 | 6 | 6 | 11 | 6 | 6 | 6 |
| F | AAG | 8 | 8 | 9 | 9 | 9 | 8 | 8 | 7 | 7 | 9 | 8 | 8 |
| G | CCA | 0 | 0 | 0 | 0 | 0 | 0 | 0 | 0 | 0 | 0 | 1 | 0 |
|  | CCC | 0 | 1 | 0 | 1 | 1 | 3 | 3 | 3 | 0 | 0 | 1 | 1 |
|  | CCG | 14 | 16 | 13 | 14 | 14 | 16 | 16 | 13 | 12 | 18 | 14 | 14 |
|  | CCU | 6 | 6 | 6 | 6 | 7 | 6 | 7 | 6 | 6 | 4 | 6 | 6 |
| H | GUG | 5 | 8 | 13 | 6 | 5 | 5 | 5 | 7 | 8 | 5 | 5 | 5 |
| I | UAA | 9 | 8 | 10 | 9 | 10 | 11 | 12 | 11 | 9 | 8 | 8 | 9 |
|  | UAG | 0 | 0 | 0 | 0 | 0 | 0 | 0 | 0 | 2 | 0 | 0 | 0 |
|  | UAU | 2 | 0 | 2 | 2 | 2 | 2 | 2 | 2 | 4 | 2 | 2 | 2 |
| K | UUC | 13 | 11 | 12 | 11 | 16 | 17 | 18 | 14 | 10 | 11 | 12 | 12 |
|  | UUU | 6 | 4 | 4 | 5 | 7 | 7 | 5 | 4 | 8 | 7 | 7 | 12 |
| L | GAA | 4 | 5 | 5 | 5 | 5 | 5 | 7 | 5 | 4 | 4 | 3 | 3 |
|  | AAC | 4 | 4 | 4 | 4 | 4 | 4 | 4 | 3 | 7 | 4 | 5 | 5 |
|  | GAC | 8 | 9 | 8 | 7 | 8 | 10 | 10 | 10 | 9 | 7 | 6 | 7 |
|  | GAG | 0 | 0 | 0 | 0 | 0 | 0 | 0 | 0 | 1 | 0 | 0 | 0 |
|  | AAU | 4 | 3 | 4 | 3 | 5 | 2 | 2 | 2 | 3 | 2 | 2 | 2 |
|  | GAU | 2 | 2 | 2 | 2 | 2 | 2 | 2 | 2 | 2 | 2 | 2 | 3 |
| M | UAC | 6 | 7 | 9 | 6 | 9 | 7 | 5 | 5 | 9 | 5 | 6 | 7 |
| N | UUG | 10 | 7 | 9 | 8 | 11 | 9 | 13 | 9 | 10 | 7 | 8 | 7 |
| P | GGA | 7 | 6 | 7 | 5 | 6 | 4 | 4 | 6 | 7 | 4 | 6 | 4 |
|  | GGC | 5 | 5 | 5 | 5 | 5 | 8 | 4 | 4 | 4 | 5 | 3 | 3 |
|  | GGU | 5 | 5 | 5 | 6 | 8 | 4 | 6 | 6 | 5 | 4 | 5 | 5 |
| Q | GUC | 8 | 8 | 8 | 8 | 8 | 8 | 7 | 7 | 6 | 12 | 7 | 7 |
|  | GUU | 4 | 4 | 3 | 5 | 6 | 6 | 5 | 11 | 6 | 4 | 4 | 12 |
| R | GCA | 10 | 9 | 9 | 9 | 12 | 12 | 10 | 13 | 10 | 9 | 10 | 10 |
|  | GCC | 0 | 0 | 0 | 0 | 0 | 0 | 0 | 0 | 1 | 0 | 0 | 0 |
|  | GCU | 10 | 5 | 7 | 7 | 7 | 8 | 4 | 5 | 7 | 4 | 5 | 5 |
|  | UCC | 3 | 4 | 4 | 4 | 4 | 2 | 5 | 4 | 5 | 2 | 2 | 2 |
|  | UCU | 3 | 3 | 3 | 3 | 3 | 3 | 3 | 2 | 4 | 3 | 2 | 2 |
| S | AGA | 9 | 8 | 7 | 9 | 9 | 6 | 11 | 9 | 8 | 6 | 6 | 6 |
|  | AGC | 4 | 3 | 5 | 4 | 5 | 7 | 3 | 5 | 3 | 4 | 3 | 4 |
|  | AGG | 0 | 0 | 0 | 0 | 0 | 0 | 0 | 0 | 1 | 0 | 0 | 1 |
|  | AGU | 2 | 2 | 2 | 2 | 2 | 3 | 2 | 2 | 3 | 2 | 2 | 2 |
|  | UCG | 6 | 3 | 6 | 6 | 6 | 8 | 5 | 5 | 6 | 3 | 5 | 5 |
| T | UGA | 8 | 8 | 9 | 8 | 8 | 9 | 8 | 8 | 8 | 7 | 7 | 7 |
|  | UGC | 3 | 3 | 4 | 3 | 5 | 3 | 4 | 3 | 4 | 4 | 4 | 4 |
|  | UGG | 0 | 0 | 0 | 0 | 0 | 0 | 0 | 1 | 1 | 0 | 0 | 0 |
|  | UGU | 6 | 5 | 6 | 6 | 6 | 7 | 8 | 8 | 7 | 8 | 9 | 7 |
| V | CAA | 6 | 7 | 8 | 7 | 7 | 7 | 7 | 7 | 8 | 6 | 6 | 6 |
|  | CAC | 7 | 6 | 8 | 7 | 10 | 7 | 6 | 5 | 5 | 6 | 6 | 7 |
|  | CAG | 0 | 0 | 0 | 0 | 0 | 0 | 0 | 0 | 1 | 0 | 0 | 0 |
|  | CAU | 2 | 2 | 2 | 2 | 2 | 2 | 2 | 2 | 5 | 2 | 2 | 2 |
| W | ACC | 8 | 9 | 8 | 8 | 8 | 6 | 14 | 8 | 7 | 6 | 6 | 6 |
| Y | AUG | 10 | 10 | 9 | 9 | 10 | 8 | 9 | 9 | 10 | 10 | 7 | 9 |

1. Drosophila 12 Genomes Consortium (2007) Evolution of genes and genomes on the Drosophila phylogeny. Nature 450: 203-218.
2. Bergman, C, Ardell, D, (2014): Nuclear tRNA gene predictions for 12 species in the genus Drosophila. figshare. http://dx.doi.org/10.6084/m9.figshare.1233437
