# Supplementary material for: A Nutrient-Driven tRNA Modification Alters Translational Fidelity and Genome-wide Protein Coding across an Animal Genus
Source: PLoS Biol. 2014 Dec 9;12(12):e1002015. doi: 10.1371/journal.pbio.1002015 (PMC4260829; doi:10.1371/journal.pbio.1002015)
Supplement: Listing S1 — R script of the kinetic model used to generate Figure 5 . (DOCX) [file pbio.1002015.s006.docx]

**Listing S1**: R script of the kinetic model used to generate Figure 5.

# Focal codons are NAC/NAU; in Fig. 5, these are Asn codons AAC/AAU

# Cognate focal tRNA is tRNA(QUN) or tRNA(GUN); in Fig. 5, this is tRNA-Asn(G/QUU)

# Competitor tRNA is XXX; in Fig. 5, this is tRNA-Thr(IGU)

# Near-cognate codon is NXU/NXC; in Fig. 5, this is Thr codon ACC

# tRNA that reads near-cognate codon is IYY; in Fig. 5, this is also tRNA-Thr(IGU)

# The k_ factors are first-order rate constants, with units of 1/(uM sec)

k_xxx_nac <- 0.004 # 1/(uM sec)

k_xxx_nau <- k_xxx_nac/3.2 # 1/(uM sec)

k_qun_nac <- 10 # 1/(uM sec)

k_qun_nau <- 2.5 # 1/(uM sec)

k_gun_nac <- 5 # 1/(uM sec)

k_gun_nau <- 2 # 1/(uM sec)

# tot is the total concentration of each tRNA, in micromolar (uM)

tot <- 1 # uM

# How G/Q-tRNA reads other codons

k_qun_nxc <- k_qun_nac/1e4

k_qun_nxr <- k_qun_nxc/2

k_gun_nxc <- k_qun_nxc*(k_gun_nac/k_qun_nac)

k_gun_nxr <- k_qun_nxr

# How other codons are read by their own cognates

# iyy = inosine-modified

k_iyy_nxc <- k_gun_nac/2

k_iyy_nxr <- k_iyy_nxc/10

# YYY = direct reader of xxR codons; in Fig. 4, this is tRNA-Thr(CGU)

k_yyy_nxr <- k_gun_nac/3.5

# Error rates

# In each error-rate calculation, if the total tRNA concentration is the same for each species, it cancels.

# We make this assumption for simplicity only.

e.nac <- function(q) {

k_xxx_nac/(k_xxx_nac + k_qun_nac*q + k_gun_nac*(1-q))

}

e.nau <- function(q) {

k_xxx_nau/(k_xxx_nau + k_qun_nau*q + k_gun_nau*(1-q))

}

e.nxc <- function(q) {

(k_qun_nxc*q + k_gun_nxc*(1-q))/(k_iyy_nxc + k_qun_nxc*q + k_gun_nxc*(1-q))

}

e.nxr <- function(q) {

(k_qun_nxr*q + k_gun_nxr*(1-q))/(k_yyy_nxr + k_iyy_nxr + k_qun_nxr*q + k_gun_nxr*(1-q))

}

# Rate of translation

r.nac <- function(q) {

k_xxx_nac*tot + k_qun_nac*q*tot + k_gun_nac*(1-q)*tot

}

r.nau <- function(q) {

k_xxx_nau*tot + k_qun_nau*q*tot + k_gun_nau*(1-q)*tot

}

# Constraints:

# G reads NAC > NAU: k_gun_nac>k_gun_nau

# Q reads NAC > NAU: k_qun_nac>k_qun_nau

# Q reads NAC and NAU better than G does: k_qun_nac>k_gun_nac and k_qun_nau>k_gun_nau

# Competitor reads NAC > NAU: k_xxx_nac>k_xxx_nau

# At q = 0, e(NAC) > e(NAU)

# At q = 1, e(NAU) > e(NAC)

#alpha <- 1 # Ratio of [XXX] to [T] = [G] + [Q]

# Assumption: [XXX] = [qun] + [gun] = [iyy] = [YYY]

# Confirm set of constraints we wish to satisfy.

constraints <- c(k_gun_nac>k_gun_nau, k_qun_nac>k_qun_nau, k_xxx_nac>k_xxx_nau, k_qun_nac>k_gun_nac, k_qun_nau>k_gun_nau, e.nac(1)<e.nau(1), e.nau(0)<e.nac(0))

stopifnot(all(constraints))

# For labeling purposes, make explicit choices for these codons.

nau <- "AAU"

nac <- "AAC"

nxc <- "ACC"

nxr <- "ACG"

q <- seq(0,1,0.01)

b <- c(e.nac(0),e.nac(1),e.nau(0),e.nau(1))

ylim <- c(min(b),max(b))

mar=c(4,4,1,1)

mult <- 1e-4

split.screen(c(2,2))

screen(1)

par(mar=mar)

# Translation error rates -- more precisely, translation error probabilities

# 'mult' specifies multiplication of the error rates (and concomitant adjustment of labels) to reduce visual clutter.

plot(q, e.nac(q)/mult, ylim=ylim/mult, lty='solid', lwd=2, type='l', log='y', las=1, ylab=paste('Error rate (x',mult,')',sep=''), xlab='Proportion of Q-modified tRNA', bg='#eee8d5')

lines(q, e.nau(q)/mult, lty='dotted', lwd=2)

text(0.2, e.nac(1)/mult, label=nac, pos=4)

text(0.1, e.nau(1)/mult, label=nau, pos=4)

screen(2)

par(mar=mar)

# Translation speeds, in amino acids per second.

plot(q, r.nac(q), lty='solid', lwd=2, type='l', log='', ylim=c(0,10), las=1, ylab='Translation speed (aa/sec)', xlab='Proportion of Q-modified tRNA')

lines(q, r.nau(q), lty='dotted', lwd=2)

text(0.9, 1.2*r.nac(0), label=nac, pos=3)

text(0.9, 1.1*r.nau(0), label=nau, pos=3)

screen(3)

par(mar=mar)

# Nonsynonymous near-cognate error rates

plot(q, e.nxc(q)/mult, ylim=c(2e-4, 4e-4)/mult, lty='solid', lwd=2, type='l', log='y', las=1, ylab=paste('Error rate (x',mult,')',sep=''), xlab='Proportion of Q-modified tRNA', bg='#eee8d5')

lines(q, e.nxr(q)/mult, lty='dotted', lwd=2)

text(0.1, median(e.nxc(0)/mult), label=nxc, pos=3)

text(0.1, median(e.nxr(0)/mult), label=nxr, pos=3)

screen(4)

par(mar=mar)

# Plot of rate constants which, assuming equal levels of each tRNA species, are proportional to rates.

# Misreading rates are multiplied by 1000 for visibility.

rates <- cbind(c(k_xxx_nau*1000, k_gun_nau, k_qun_nau),c(k_xxx_nac*1000, k_gun_nac, k_qun_nac))

barplot(rates, horiz=T, beside=T, col=rev(c('black','gray90','gray50')), log='', names.arg=c('AAU','AAC'), las=1, xlab='First-order translation rate constant (1/(\u03BCM sec))')

close.screen(all=TRUE)
